# Microbial responses to ocean alkalinity enhancement in seasonally hypoxic coastal sediments

**DOI:** 10.3389/fmicb.2026.1854407

**Published:** 2026-06-23

**Authors:** Stefanie Böhnke-Brandt, Rebecca Bährle-Wunsch, Michael Fuhr, Andrew W. Dale, Janina Fuß, Sonja Geilert, Mirjam Perner

**Affiliations:** 1 Geomicrobiology, GEOMAR Helmholtz Centre for Ocean Research Kiel, Kiel, Germany; 2 Benthic Biogeochemistry, GEOMAR Helmholtz Centre for Ocean Research Kiel, Kiel, Germany; 3 Competence Centre for Genomic Analysis (CCGA), Institute of Clinical Molecular Biology (IKMB), Kiel University, Kiel, Germany; 4 Department of Earth Sciences, Utrecht University, Utrecht, Netherlands

**Keywords:** Baltic Sea sediments, carbon dioxide removal (CDR), enhanced benthic weathering (EBW), microbial community dynamics, mineral dissolution, ocean alkalinity enhancement (OAE), seasonal hypoxia

## Abstract

**Background:**

Enhanced benthic weathering (EBW) is a promising marine carbon dioxide removal (mCDR) strategy, where alkaline minerals are added to the seafloor. Upon weathering, these artificially deposited minerals release alkalinity to the water column, which ultimately leads to CO_2_ uptake from the atmosphere. However, feedbacks of the benthic bacterial community to this mCDR approach remain unknown.

**Methods:**

To investigate the effects of EBW, sediment core incubations with added alkalizing minerals were carried out and shifts in bacterial communities identified and linked to sedimentary properties. Surface sediments with supernatant water from a seasonally hypoxic basin in the Baltic Sea off the eastern coast of northern Germany were collected and either calcite (limestone) or silicate (dunite) added and kept under oxic (avg. 263 ± 34 μM O_2_) or oxygen-limited (0 to maximum 162 μM O_2_) conditions.

**Results:**

Bacterial surface sediment communities responded to the different oxygen availabilities in the overlying bottom waters independent of the mineral addition as reflected in cluster formation via Principal Coordinates Analysis (PCoA). Although, by contrast, mineral addition induced less pronounced changes, the relative abundances of several rare taxa changed significantly, such as chemolithoautotrophs associated with the sulfur and nitrogen cycles. *Candidatus* Electrothrix was selectively promoted by calcite addition under oxygen-limited conditions in two of three cores, whereas *Nitrosomonas* exhibited increased growth tendencies following dunite addition under both oxic and oxygen depleted bottom water conditions. Sulfur oxidizing bacteria (SOB) of *Beggiatoaceae* declined under oxygenated water conditions, independent of the type of mineral added. The dunite amendment was associated with an increase in other SOB while calcite amendment led to the enrichment of organotrophic taxa.

**Conclusion:**

Our findings provide important insights into the response of microbial communities to EBW, but also highlight the necessity of field-based studies to accurately assess the effects of EBW on coastal ecosystems.

## 1 Introduction

Atmospheric carbon dioxide (CO_2_) levels have been increasing since the industrial revolution, resulting in warming of Earth’s climate (Hansen et al., 2010; Howe, 2015; MacFarling Meure et al., 2006; Keeling et al., 2001; Petit et al., 1999; Valipour et al., 2021). The oceans are fundamental in helping to slow down climate change by taking up heat and CO_2_ from the atmosphere (DeVries et al., 2017; Gruber et al., 2023). This has caused rapid changes of the oceans including higher water temperatures, deoxygenation and acidification, in turn affecting oceanic life and biogeochemical cycles (Schmidtko et al., 2017; Brauko et al., 2020; Breitburg et al., 2018). To mitigate or even counteract anthropogenically induced changes to Earth’s climate, CO_2_ must be actively removed from the atmosphere and stored over long time scales (IPCC, 2022). One of the currently tested marine CO_2_ removal (mCDR) strategies is ocean alkalinity enhancement (OAE) (Gattuso et al., 2018; Kheshgi, 1995) via enhanced benthic weathering (EBW) (Dale et al., 2024; Fuhr et al., 2023; Fuhr et al., 2024; Fuhr et al., 2025). In EBW, minerals like calcite (calcium carbonate) or silicate (dunite rock) are added to marine surface sediments, where their dissolution generates alkalinity (A_T_) and stimulates CO_2_ uptake of the water body (Dale et al., 2024; Fuhr et al., 2023; Meysman and Montserrat, 2017; Montserrat et al., 2017; Fuhr et al., 2025; Fuhr et al., 2024).

The Baltic Sea features regions where bottom waters are exposed to seasonal or permanent oxygen depletion. These temporally or permanently calcite-undersaturated deep waters offer favorable conditions for achieving most efficient EBW rates (Dale et al., 2024; Fuhr et al., 2023; Fuhr et al., 2024). Economic evaluations indicate that near coastal regions are more viable for large-scale CDR strategies compared to the open ocean (Fuhr et al., 2025; Köhler et al., 2013; Renforth and Henderson, 2017). Bottom waters at the shallow coastal Boknis Eck site in Eckernförde Bay (SW Baltic Sea, Germany) are regularly exposed to seasonal hypoxia to anoxia in late summer (Alampalli et al., under review; Perner et al., 2022). Due to these recurring low-oxygen conditions, Boknis Eck is an ideal natural laboratory to evaluate the effectiveness of EBW as a CDR strategy and to investigate potential environmental implications in a realistic temperate costal setting (Fuhr et al., 2023, 2024). Boknis Eck sediments are rich in organic matter (~5 wt.%) (Dale et al., 2013; Wallmann et al., 2022) and contain up to 4 wt.% Fe-sulfide minerals, i.e., iron mono-sulfide (FeS) and pyrite (FeS_2_) (Perner et al., 2022). Carbonate distribution of surface sediments in the region is spatially patchy (0.5 to 7 wt.%) (Wallmann et al., 2022; Dale et al., 2013). The undersaturation of calcite in the sediments of Boknis Eck reaches a depth of 0.4 cmbsf in summer and 1.3 cmbsf in winter (Dale et al., 2024). This is primarily driven by elevated rates of acidifying aerobic respiration in winter, when bottom waters are well-ventilated, and reduced aerobic rates in summer under oxygen-depleted conditions. These seasonal variations are accompanied by changes in bottom water carbonate chemistry. While the natural variation in A_T_ is rather small (1.8 mmol L^−1^ – 2.1 mmol L^−1^), the accumulation of metabolic CO_2_ leads to undersaturation with respect to aragonite and calcite during times on hypoxia and anoxia (Melzner et al., 2013).

Possible feedback mechanisms of the benthic microbial community to the addition of alkalizing minerals for EBW under varying oxygen availabilities have so far not been investigated. In this study, sediment cores from Boknis Eck were incubated in the dark under oxic and low-oxygen conditions after depositing alkaline minerals (calcite and dunite) to the sediment surfaces. Shifts in the composition and function of benthic microbial communities and associated biogeochemical cycling were monitored over the course of the experiments. Microbial data were combined with EBW-related chemistry changes in the water body and sediment porewaters, to identify taxa showing significant correlation with the observed geochemical shifts. Our results show distinct changes in benthic microbial communities, providing first insights into ecosystem-scale implications of EBW.

## 2 Materials and methods

### 2.1 Description of the study site

Boknis Eck is a well-studied coastal site in the southwestern Baltic Sea (10°2,46′E, 54°31,8’N) that is subject to pronounced seasonal bottom-water oxygen fluctuations (Melzner et al., 2013). The site experiences regular transitions between oxic and anoxic conditions, making it an ideal natural laboratory to investigate microbial and biogeochemical dynamics under changing redox regimes (Alampalli et al., under review; Perner et al., 2022). Following the spring phytoplankton bloom, enhanced export of organic matter to the seafloor stimulates aerobic respiration by benthic microbes, thereby increasing oxygen demand in surface sediments (Hepach et al., 2024; Lennartz et al., 2014). Together with rising surface-water temperatures and the onset of water column stratification, this enhanced respiration drives progressive oxygen depletion in bottom waters (Hansen et al., 1999). Typically, anoxic conditions develop by late summer and persist until winter storms disrupt stratification and reintroduce oxygenated surface waters to deeper layers (Lennartz et al., 2014; Dietze and Löptien, 2021).

### 2.2 Experimental design and sampling description

**Figure 1:**
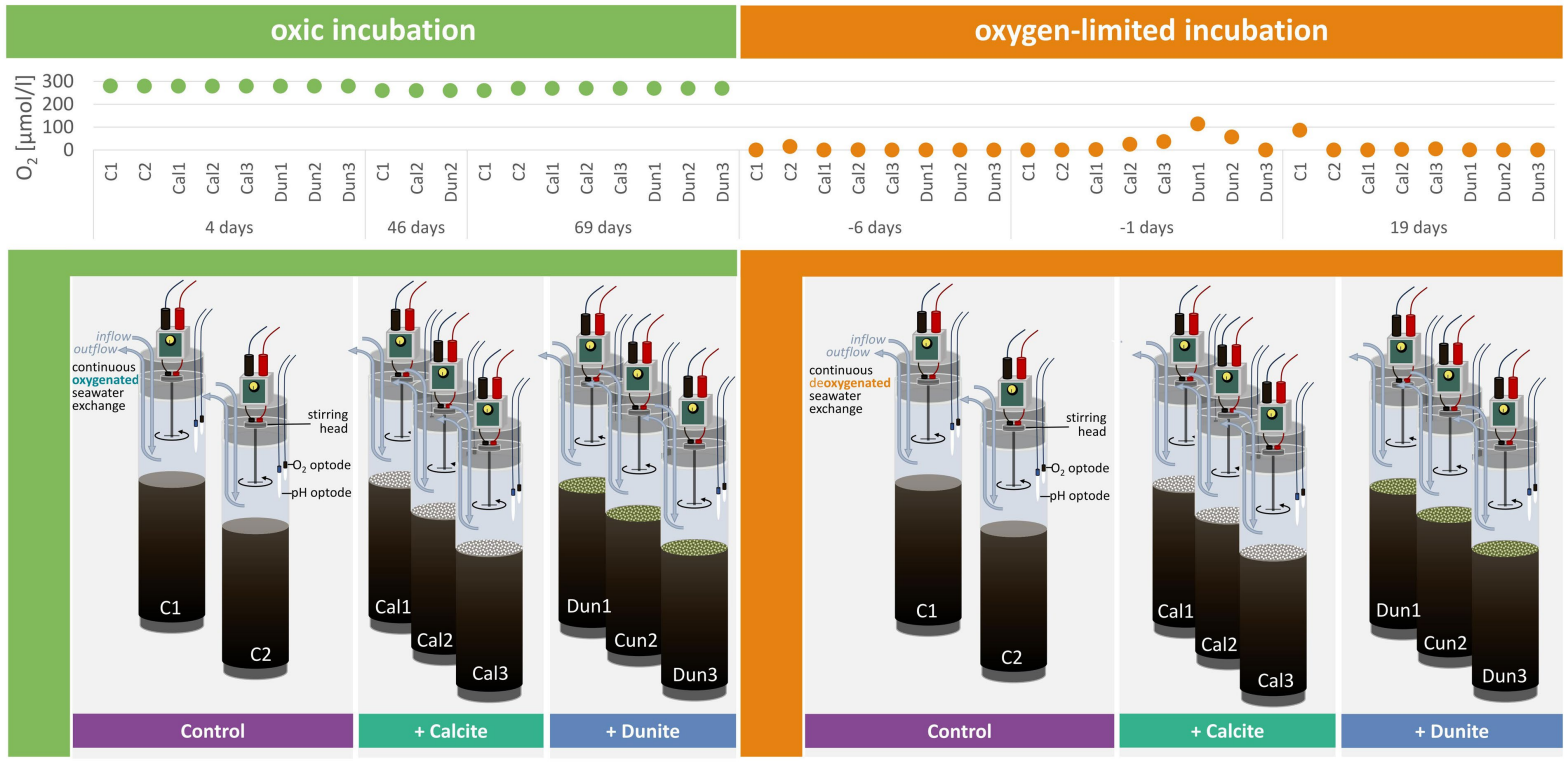
Experimental setup and oxygen dynamics during sediment core incubations under oxic and oxygen-limited conditions. Sediment cores from Boknis Eck were incubated in the dark to assess microbial and biogeochemical responses to the addition of alkalizing minerals (calcite and dunite). The upper panel shows oxygen concentrations in bottom waters around the time of microbial subsampling. Sampling time points are given relative to the day of mineral addition.

The experimental setup of the oxic and oxygen-limited sediment core incubations has been described in detail elsewhere (Fuhr et al., 2024; Fuhr et al., 2023). Here, we provide a brief overview, with a focus on the aspects relevant to microbial community dynamics (Figure 1).

The benthic microbial communities at the sampling site are known to reflect seasonal hypoxic environmental endpoint conditions (Alampalli et al., under review; Perner et al., 2022). In order to mirror these seasonally adapted bacterial communities, the incubations aimed at simulating oxygenated bottom waters in late winter and oxygen-depleted deep waters in early fall. Eight sediment cores were retrieved for each of the incubation conditions (oxic and oxygen-limited) and kept in the dark at 12.5 °C, reflecting typical summer bottom-water temperatures at the natural site (Melzner et al., 2013). Cores for the oxic incubation were sampled during the well mixed winter period on 13 January 2022, when bottom waters were fully oxygenated (oxygen = 243 μM), following seasonal re-ventilation of the water column (Alampalli et al., under review; Fuhr et al., 2023). In contrast, cores for the oxygen-limited incubation were collected on 26 October 2022, approximately 4 weeks after bottom waters at Boknis Eck were anoxic (oxygen <1 μM) and coincided with the onset of re-ventilation of the deep waters reaching an oxygen concentration of 179 μM (Alampalli et al., under review; Fuhr et al., 2024).

Immediately after retrieval, cores were sealed with rubber stoppers, transported to the incubation facility and prepared for incubation on the same day. Sediment cores were subjected to three treatments: (i) unamended control (C1, C2), calcite addition (Cal1, Cal2, Cal3) and dunite addition (Dun1, Dun2, Dun3). Due to technical limitations of the experimental setup (limited pump capacity), the unamended control treatment was conducted in duplicate, whereas amended treatments were conducted in triplicate. The amount of added dunite and calcite was based on the rain rate of particulate organic carbon measured at Boknis Eck, equaling 0.5 mmol cm^−2^ a^−1^ (Dale et al., 2011) and then doubled to obtain a measurable weathering signal (Fuhr et al., 2024). Thus, in the mineral amended sediment incubations 7.86 g limestone calcite; Cal1, Cal2 and Cal3 or 4.48 g olivine AFS 80 dunite; Dun1, Dun2 and Dun3 were evenly distributed on the sediment surfaces. Mineral compositions for dunite were ~89% olivine, ~7% pyroxene, ~2% serpentinite and others <2% and for limestone ~98% calcite. Average grain size distribution of the washed calcite material was 87 μm and of the dunite 20–30 μm (Fuhr et al., 2023).

For the oxic incubation stirring heads with continuous oxygenated-water exchange were installed after minerals were added The cores were then left to equilibrate until the first subsampling event of the 0–2 cm sediment horizon, which took place 4 days after mineral addition (t0_oxic_ = day 4) to capture the initial community composition and chemical settings in all eight cores. A second subsampling was conducted 46 days after mineral addition (t2_oxic_ = day 46), when one core per treatment (C1, Cal2 and Dun2) was subsampled. The final sampling occurred 69 days after mineral addition (t3_oxic_ = day 69).

For the oxygen-limited incubation, sediment cores were kept sealed for 7 days until the thin oxidized surface layer became anoxic. First subsamples from all eight oxygen-limited incubations were then taken from the top 2 cm of sediment (t0_oxygen-limited_ = −6 days). Then stirring heads were installed for logging of pH and oxygen in waters. Sediment core waters were continuously exchanged with Boknis Eck water bubbled with dinitrogen (N_2_) gas (flow rate 700 μL min^−1^). Although the gas phase above the water column was kept as small as possible, oxygen occasionally invaded the waters in the different sediment core incubation experiments. The overall oxygen invasion was due to technical limitations, whereby two peristaltic pump channels had to be used for continuous exchange of deoxygenated water of each individual treatment (1: inlet, 2: outlet). If the peristaltic pump channels did not run 100% synchronously (due to, e.g., different deformation of the tubing, or minimal manufactural differences), a minimal under-pressure could develop that sucked in ambient air and caused oxygen invasion over time. The second set of subsamples was taken 6 days after stirring heads were installed, just 1 day before mineral addition (t2_oxygen-limited_ = −1 day) and the third subsampling was performed after 19 days (t3_oxygen-limited_ = 19 days). Subsamples were collected with a custom syringe designed to minimize sediment-surface disturbance and maintain experimental integrity (Fuhr et al., 2023). Portions of the subsamples were preserved and stored for microbiological analyses according to the procedures outlined in the methods sections below.

### 2.3 Quantitative analyses: cell numbers and CARD-FISH

For total cell counts via diamidino-2-phenylindole (DAPI) and catalyzed reporter deposition–fluorescence *in situ* hybridization (CARD-FISH) subsamples of the 0–1 cm and 1–2 cm sediment horizons from the eight oxic and eight oxygen-limited cores were fixed in 4% formaldehyde/phosphate buffered saline (PBS) overnight at 4 °C. After washing the samples twice with sterile PBS buffer, they were stored at −20 °C in PBS/EtOH [1:1] until further processing. Cells were then detached from the sediment by ultrasonication (2 times: 20%, 20 cycles, 20 s) and diluted with sterilized PBS [1:4000] before they were concentrated onto polycarbonate filters (0.2 μm pore size, Nucleopore, Whatman, UK) using vacuum filtration (−200 mbar). Cell enumeration was carried out using a Zeiss Axio Imager. M2 epifluorescence microscope, equipped with EGFP 214 and DAPI filter sets. For cell imaging, a Zeiss Axio Cam MRM. Rev2 in combination with the 215 ZEN 2.3 software package was used. At least 500 cells or 20 grid squares were counted per sample to ensure statistical robustness.

CARD-FISH analyses to further investigate cable bacteria morphology were conducted following the procedure described by Pernthaler et al. (2002) and Thiele et al. (2011) with minor modifications. Briefly, filters were embedded in 0.2% Metaphor agarose, and filter sections designated for bacterial cell permeabilization were incubated with lysozyme solution (10 mg ml^−1^ lysozym, 0.5 M EDTA, 1 M Tris–HCl, pH 8) at 37 °C for 60 min, followed by treatment with achromopeptidase solution (180 U ml^−1^ achromopeptidase, 0.01 M NaCl-Tris buffer, pH 8) at 37 °C for 15 min. Subsequent inactivation was carried out in 0.01 M HCl solution for 10 min at room temperature (22 °C). Hybridization was performed overnight at 35 °C in hybridization buffer (0.9 M NaCl, 0.02 M Tris–HCl pH 7.4, 10% blocking reagent, 2% w/v dextran sulfate, 0.01% SDS, 35% formamide) including the probe DSB706 diluted 1:1510. This probe targets most members of the *Desulfobulbaceae*, including cable bacteria as well as species (Loy et al., 2002). For signal amplification, filters were incubated in tyramide mix at 37 °C for 30 min. Filters were subsequently counterstained with DAPI for 10 min at room temperature. The NON-EUB probe (Wallner et al., 1993) labeled with FITC was used as a negative control to verify the absence of non-specific binding.

### 2.4 Generating and processing of 16S rRNA gene amplicons from RNA

Frozen sediments (−80 °C) from the top 2 cm of the unamended control (C1, C2), the calcite (Cal1, Cal2, Cal3) and the dunite (Dun1, Dun2, Dun3) treatments for the three subsampled timepoints in the oxic (t1-t3_oxic_) and oxygen-limited (t1-t3_limited-oxygen_) incubation were used for RNA isolation, reverse-transcribed into cDNA and used to generate 16S rRNA gene amplicons. RNA was isolated from approximately 500 mg sediment sample using the NucleoBond RNA Soil Mini Kit (Macherey-Nagel, Düren, Germany). Subsequently, DNase I digestion was performed applying the RapidOut DNA Removal kit (Thermo Fisher Scientific GmbH, Waltham, MA, USA). Extracted RNA was reverse transcribed into cDNA according to the Invitrogen SuperScript® VILO™ cDNA Synthesis Kit (Life Technologies™, Darmstadt, Germany) and immediately purified (Zymo Clean & Concentrator-5, Irvine, CA, USA). For amplicon library construction the hypervariable V3 and V4 regions of the reverse transcribed bacterial 16S rRNA gene were amplified using primer pairs S-D-Bac-0341-b-S-17 and S-D-Bac-0785-a-A-21 for bacterial 16S tags (Klindworth et al., 2013). Sequencing was done in collaboration with the Competence Centre for Genomic Analysis (CCGA, Kiel, Germany) in a 2 × 300 bp paired-end sequencing run on the Illumina MiSeq platform (Illumina, St. Diego, USA) as described before (Böhnke et al., 2019, Perner et al., 2022).

Processing of amplicon data was done in a Qiime2 environment (Bolyen et al., 2019). Filtering and merging of demultiplexed raw reads were conducted using the dada2 plugin with default settings and removal of the previous primer sequences (Callahan et al., 2016). The SILVA database release 138 (Quast et al., 2013) was pre-trained with the respective primer pairs for bacteria (Pedregosa et al., 2011; Bokulich et al., 2018) to obtain taxonomic assignments, which were computed using the feature-classifier plugin (classify sklearn) with default settings (Bokulich et al., 2018). The phylogeny was calculated using the align-to-tree-mafft-fasttree pipeline (Chistoserdova, 2010).

### 2.5 Data analysis and statistical methods

Data analysis, statistics and visualizations were performed using R software (R Foundation for Statistical Computing, Vienne, Austria. URL^1^) with the “microeco” package (Liu et al., 2021). Prior to downstream analyses the dataset was filtered to retain bacterial sequences only, and remove non-target taxa including mitochondria, chloroplasts, Archaea, and Eukaryota. Samples were then rarefied to the lowest sequencing depth (=31,500 reads per sample) before calculating relative abundances and statistics.

Alpha diversity was assessed using the Shannon diversity index, and pairwise comparison among treatments were performed using two-sided Wilcoxon rank-sum tests with no *p*-value adjustment. Beta diversity was evaluated based on weighted and unweighted UniFrac distances and visualized by Principle Coordinate Analysis (PCoA). The ellipses were calculate based on the treatment, i.e., control, calcite and dunite in all plots. Spearman rank correlations were performed to evaluate associations between relative abundances of bacterial 16S rRNA gene amplicons and environmental parameters including A_T_, O_2_, B, Mn, Ca^2+^, Fe, Na, Mg, Sr., Si, Ba, Li, K, DIC, pH and H_2_S. The corresponding geochemical data are provided in Supplementary Table S1 and have been published and described in detail in Fuhr et al. (2023, 2024). The correlation was done with p-adjust method = “none” and correlation methods = “spearman” (Benjamini and Hochberg, 1995) on the genus level. Differential abundance analysis (DAA) was performed using the linear discriminant analysis (LDA) effect size (LEfSe) method (Segata et al., 2011), with no p-value adjustment. Taxa were ranked according to their LDA scores, which reflect the magnitude of their contribution to the differentiation between groups. Higher LDA scores indicate a greater effect size and stronger discriminatory power.

### 2.6 Geochemical analysis

Bottom water samples (12 mL) were collected from the outflow of each core over several minutes, filtered through 0.2 μm cellulose membrane filters, and stored refrigerated until analysis. Sampling frequency decreased from daily during the first 2 weeks to every 2–4 days toward the end of each experiment. Total A^T^ was determined by titration of 1 mL sub-samples with 0.02 N HCl following Ivanenkov and Lyakhin (1978), with continuous nitrogen degassing to remove CO₂ and H₂S, and standardized against an IAPSO seawater standard. Anion concentrations (SO₄^2−^, Cl^−^, Br^−^) were measured by ion chromatography (IC; METROHM 761 Compact, conductivity mode). Acidified sub-samples (3 mL + 30 μL suprapure HNO₃) were analyzed for major and trace elements (Si, Na, K, Li, B, Mg, Ca, Sr., Mn, Ni, Fe) by inductively coupled plasma optical emission spectroscopy (ICP-OES; Varian 720-ES). In the oxic experiment (Fuhr et al., 2023), nutrients (NH₄^+^ by manual photometry; PO₄^3−^, NO₂^−^, NO₃^−^ by Seal-Analytical™ QuAAtro autoanalyser) were additionally measured from frozen 5 mL aliquots.

At the end of each experiment, cores were sliced and pore waters recovered by centrifugation (50 mL Falcon tubes, 3,000 rpm, 10 min), filtered (0.2 μm), and transferred to polyethylene vials under oxygen-free conditions. Dissolved Fe^2+^ was stabilized with ascorbic acid and quantified by Ferrozin complexation within 30 min; H₂S was fixed with zinc acetate gelatin solution following dilution with oxygen-free artificial seawater (Dale et al., 2014; Dale et al., 2016). Freeze-dried and ground solid phase samples were analyzed by flash combustion for total carbon (TC), total organic carbon (TOC), total nitrogen (TN), and total sulfur (TS) — using a Carlo Erba NA-1500 (oxic experiment) or a EuroEA 3,000 (EuroVector; anoxic/hypoxic experiment). Total inorganic carbon (TIC) was calculated as TC − TOC, with method accuracy verified using BBOT and an internal sediment standard.

Bottom water pH and O₂ were continuously recorded using Pyroscience™ optodes (PHROBSC-PK8 and OXROB3-SUB). In the anoxic/hypoxic experiment, a self-constructed 16-channel logging device (PICO-O2-SUB and PICO-PH-SUB, Pyroscience) was used. All pH optodes and micro-sensors were calibrated using three TRIS buffers for brackish water conditions (Pratt, 2014; Müller et al., 2018); pH is reported on the total scale (pH_T_; Dickson, 1993). Sediment micro-profiling was carried out with a motorised Unisense™ micromanipulator using Clark-type micro-sensors for O₂ (Ox-50; 100 μm steps), pH (pH-100; 300 μm steps), and H₂S (SULPH-100; 300 μm steps; calibration at 109 μmol L^−1^). Total dissolved sulfide concentrations were calculated from combined H₂S and pH profiles following Millero et al. (1988) and Jeroschewski et al. (1996). Carbonate system properties (DIC) were calculated from measured pH and A_T_ following Zeebe and Wolf-Gladrow (2001), using equilibrium constants appropriate for the respective salinity, temperature, and pressure.

## 3 Results

### 3.1 Chemical dynamics of oxic vs. oxygen-limited treatments

**Figure 2:**
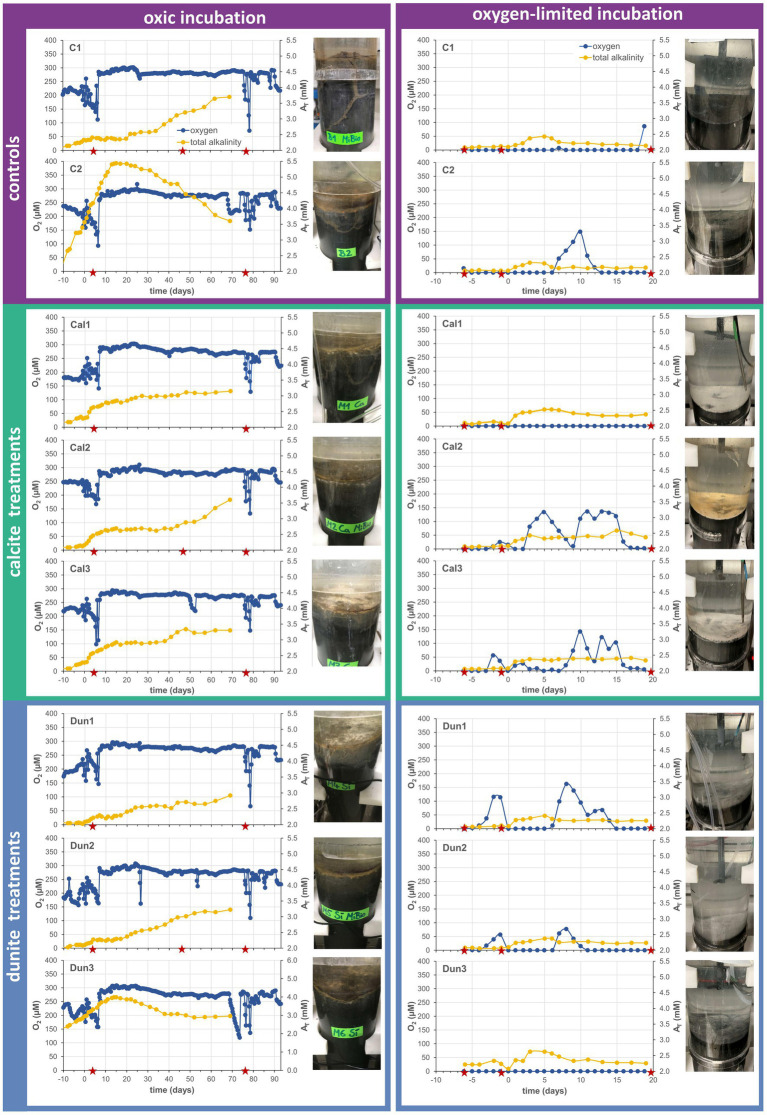
Oxygen concentration (blue line) and total alkalinity (A_T_ yellow line) in bottom water during oxic (left) and oxygen-limited (right) incubations data from (Fuhr et al., 2023, 2024) re-sketched. The top panel shows control treatments C1 and C2 (purple edged), the middle panel shows calcite treatments Cal1, Cal2, and Cal3 (green edged), and the bottom panel shows dunite treatments Dun1, Dun2, and Dun3 (blue edged). Time (days) is given relative to the day of mineral addition (day 0). Red stars mark the time points of microbial subsampling. A photo of the corresponding sediment core, taken at the end of the experiment with focus on the sediment surface, is shown next to each diagram.

Detailed chemical characterizations of the incubations, i.e., pore- and bottom-water chemistry, solid-phase composition, and sediment-water fluxes, have been reported in Fuhr et al. (2023) for oxic and in Fuhr et al. (2024) for oxygen-limited treatments. Here, we provide a brief summary focusing on oxygen and A_T_ to contextualize the microbial responses in relation to oxic and oxygen-depleted water in the core incubation (Figure 2).

In the oxic incubations, oxygen concentration remained largely stable between 270 and 300 μM across all eight sediment cores, with two brief technically induced decreases: one shortly after mineral addition and another between days 70 and 80. Neither decrease in oxygen, however, resulted in hypoxic water conditions (Figure 2).

In contrast, under oxygen limited water conditions, the reproducibility of oxygen levels was more challenging. Of the oxygen-limited treatments, Cal1 and Dun3, oxygen concentrations remained persistently below 2 μM. Other sediment cores experienced episodic entrainment of oxygen, up to 148 μM in C1, 136 μM in Cal2, 142 μM in Cal3, 162 μM in Dun1, and 77 μM in Dun2, yet microoxic to anoxic water conditions dominated the overall incubation period (Figure 2). By the end of the experiment on day 19, oxygen concentrations of overlying waters had declined to microoxic levels (<3 μM) in all cores, except for a brief late-stage rise to 86 μM in C1.

Clear differences in sediment surface features were observed after exposure to oxic and oxygen-limited waters (Figure 2). Under oxic conditions, the sediment surfaces showed pronounced signs of bioturbation and meiofaunal activity, resulting in particle mixing and downward transport of the added minerals into deeper sediment layers, reaching down to ~1.5 cm sediment depth (Fuhr et al., 2023). In contrast, sediments from the oxygen-limited incubation showed no visible signs of bioturbation and the minerals persisted as a distinct, unmixed layer on the sediment surface throughout the experiment. The sediments in the oxygen-limited incubation appeared much darker, likely driven by Fe-sulfide formation which is characteristic for these anoxic sediments (Zhou et al., 2023; Perner et al., 2022). Notably, the Cal2 core in the oxygen-limited incubation, which experienced the longest phase of oxygen intrusion (day3 to day16), showed a distinct rust-brown coloration of the sediment surface (Figure 2) indicative of iron (oxyhydr)oxide formation (Emerson et al., 2010).

In the oxic incubations, alkaline mineral additions induced a rapid increase of total A_T_ in calcite (Cal1, Cal2, and Cal3) and two of three dunite (Dun1 and Dun2) treated cores. This initial mineral driven A_T_ rise leveled off within a few days, after which A_T_ continued to increase gradually, likely due to ongoing dissolution of added minerals along with natural alkalinity generating processes, such as pyrite burial and natural weathering. Towards the end of the experiments (day 69) A_T_ values peaked between 2.9 mM to 3.6 mM. In contrast, Dun3 exhibited an A_T_ increase prior to mineral addition, showing an early peak of 4 mM between days 14 and 17, followed by a decline to 3.0 mM by the end of the experiment. Untreated cores C1 and C2 displayed substantial A_T_ rise despite no mineral additions: C1 increased more gradually, reaching a peak of 3.7 mM at the end of the experiment, while C2 showed an early peak between days 14 and 17 of 5.4 mM, similar to Dun3 A_T_ pattern, and declined to 3.6 mM, which was in the upper range of values observed in mineral-treated cores. Under oxygen-limited conditions, A_T_ concentrations were generally low compared to the oxic treatments, reflecting the minimal activity of natural A_T_-generating processes, as evident from the A_T_ patterns observed in the control cores C1 and C2. Unlike in the oxic incubation, calcite treatments, showed a significant increase over the course of the experiment relative to both controls (two-sided unpaired Student’s t-test, C1: *p*-values = 0.001, 0.003 and 0.006 for Cal1 - Cal3; C2: p-values = 1.4 · 10^−5^, 3.4 · 10^−5^ and 2.1 · 10^−5^ for Cal1-Cal3). In dunite treated cores A_T_ concentration also increased in overlaying water. However, the observed increase was only significant relative to C2 (<0.005) but not to C1. A clearer picture emerges when examining the A_T_ efflux data: calcite treated sediment cores incubated under oxygen-limited conditions exhibited the highest benthic A_T_ efflux (Supplementary Figure S1). The addition of calcite enhanced benthic A_T_ release by 2.94 μmol cm^−2^ d^−1^, while dunite addition increased fluxes by 1.12 μmol cm^−2^ d^−1^ relative to the unamended controls (Fuhr et al., 2024). In contrast, experiments conducted under oxic water conditions resulted in lower A_T_ fluxes, namely 0.91 μmol cm^−2^ d^−1^ for calcite and 0.22 μmol cm^−2^ d^−1^ for dunite, with calcite showing a more pronounced effect than dunite, similar to what was observed in the oxygen-limited incubations.

### 3.2 Cell abundance

**Figure 3:**
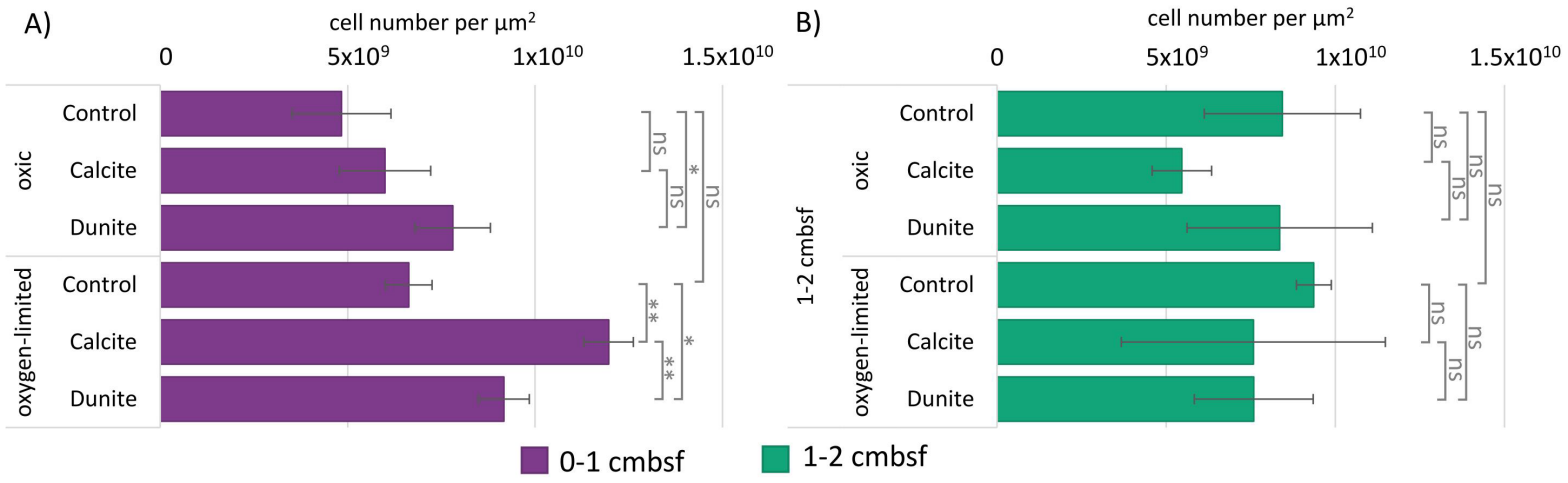
Total cell numbers based on DAPI are shown for the two investigated sediment layers **(A)** 0–1 cmbsf and **(B)** 1–2 cmbsf. Standard deviation based on biological replicates. Statistical significance is indicated by asterisks (*p ≤ 0.05; **p ≤ 0.01; ***p ≤ 0.001; ns = not significant). Total cell counts represent the sum of cells recovered from pore water and cells detached from sediment particles via ultrasonication.

Mean cell numbers were calculated across replicates, to identify overarching patterns (Figure 3). However, it should be noted that there is a natural heterogeneity among parallel sediment cores within the same treatment. Individual sediment core data for all replicates within one treatment are shown in Supplementary Figure S2. Overall, cell numbers consistently range between 10^9^ and 10^10^ cells per μm^2^, regardless of treatment, which is in line with previous observations for Boknis Eck sediments (Perner et al., 2022). In the controls, total cell numbers for the two investigated sediment depth layers (0–1 cmbsf and 1–2 cmbsf) were 4.8·10^9^ ± 1.2·10^9^ and 8.4·10^9^ ± 2.3·10^9^ cells per μm^2^ for the oxic incubation, and 6.6·10^9^ ± 2.2·10^8^ and 9.4·10^9^ ± 5.1·10^8^ cells per μm^2^, for the oxygen-limited incubation, respectively are estimated. Despite seasonal differences in sampling, these values did not differ on a level of significance. Calcite addition under oxic incubation conditions did not result in significant changes in absolute cell abundances in either the 0–1 cmbsf layer or the 1–2 cmbsf layer. By contrast, under oxygen-limited conditions, the addition of calcite led to a significant increase in cell numbers in the 0–1cmbsf layer. Yet, in the sediment horizon 1–2 cmbsf calcite did not cause any significant change in cell numbers, although it is worth noting that there was pronounced variability among replicates (Cal1 = 4.6·10^9^ cells per μm^2^, Cal2 = 6.2·10^9^ cells per μm^2^, Cal3 = 1.2·10^10^ cells per μm^2^, see Supplementary Figure S2), likely masking possible treatment effects. Dunite amendment under oxygen-limited conditions resulted in a significant increase in absolute cell abundances at both investigated sediment depths.

### 3.3 (Dis)similarity of bacterial community composition and diversity

Shannon diversity was assessed to evaluate within-sample bacterial diversity (Supplementary Figure S3). Across all sediment samples, Shannon diversity averaged 6.56 ± 0.20 (mean ± SD; *n* = 43). No significant differences in alpha diversity were observed between oxic and oxygen-limited incubations (Wilcoxon rank-sum test, *n* = 43, *p* = 0.34) (Supplementary Figure S3A). Likewise, no significant treatment effects were detected within the two incubation conditions, as Shannon diversity did not differ significantly between control and mineral-amended sediments (all Wilcoxon rank-sum tests, *p* > 0.05) (Supplementary Figures S3B,C).

**Figure 4:**
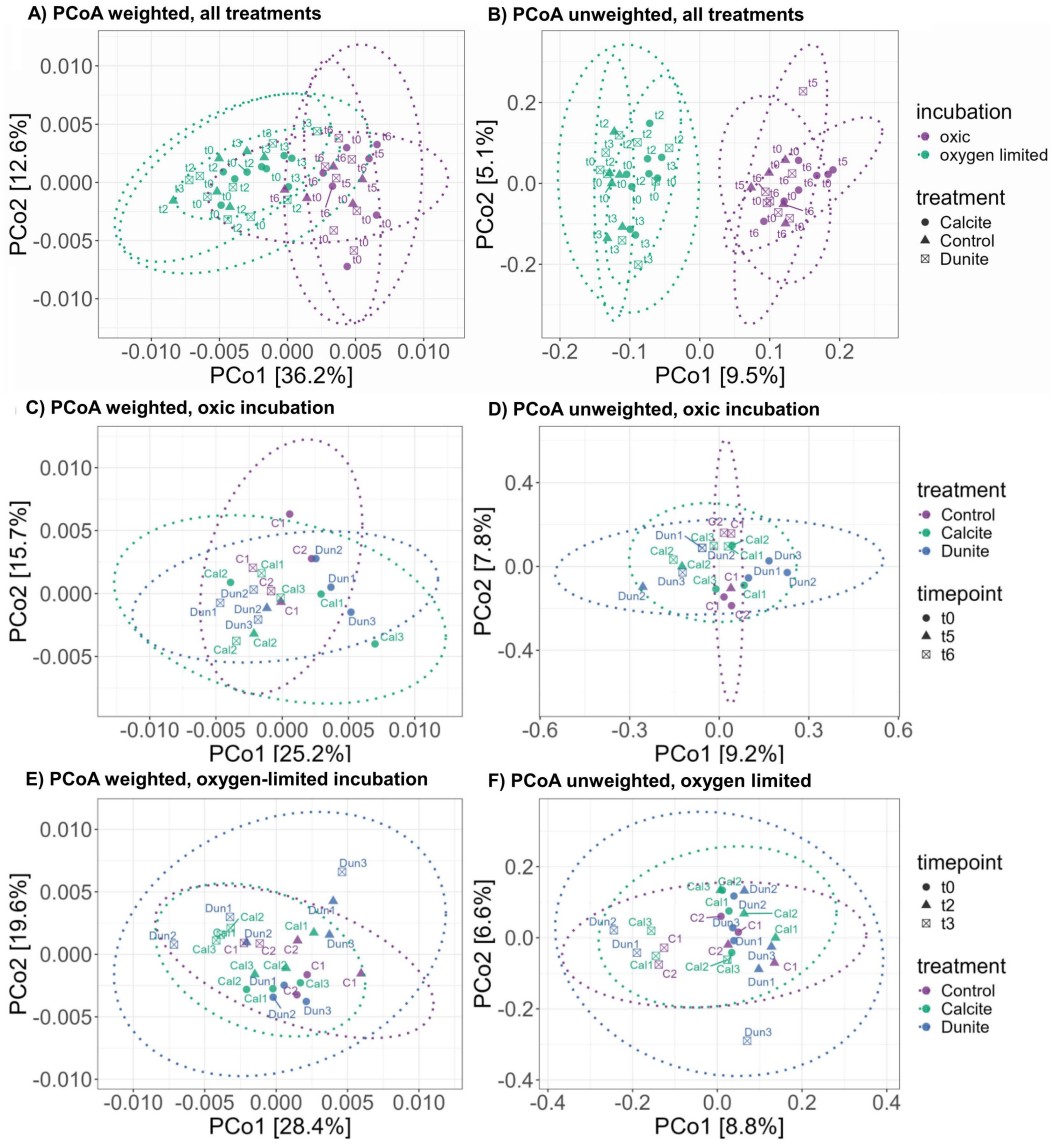
Principal Coordinates Analysis (PCoA) plots of bacterial communities in the top 2 cm across different treatments. Plots show **(A,C,E)** weighted and **(B,D,F)** unweighted Unifrac distances of all treatments and timepoints **(A,B)** and within subsets of samples from oxic- **(C,D)** and oxygen-limited **(E,F)** incubations. Samples time points for the oxic experiment were t0 = 7 days before mineral addition, t2 = 1 day before mineral addition, and t3 = 21 days after mineral addition. For the oxygen-limited incubation samples correspond to t0 = 4 days after mineral addition, t2 = 46 days after mineral addition and t3 = 76 days after mineral addition. The ellipse was calculate based on the treatment, i.e., control, calcite and dunite in all plots.

Despite the comparable alpha diversity across treatments and incubation conditions, beta diversity analyses revealed a clear separation of benthic bacterial communities driven by seasonally controlled oxygen availability and temperature effects in the overlaying bottom water, distinguishing fully oxic, cold (7 °C at 28 m depth) February samples from oxygen-limited, warmer (13 °C at 28 m depth) October samples in both untreated and mineral-treated sediment cores (Figures 4A,B).

Although the degree of clustering differed between weighted and unweighted UniFrac distance algorithms (Figures 4A,B). While the unweighted PCoA resulted in a distinct grouping of samples from February and October sediment core incubations (Figure 4B), the weighted PCoA gave less pronounced clusters exhibiting some overlap (Figure 4A). These patterns suggest that the control and both mineral treatments share abundant bacterial taxa, as reflected by their proximity in the weighted PCoA (Figure 4A), while differences in low abundant taxa contribute to the greater separation observed in the unweighted PCoA (Figure 4B). Given that the sum of the percentages of the first two principle coordinates, A (9.5%) and B (5.1%), in the unweighted PCoA is less than 15% (Figure 4B), it can be concluded that the axes explain only a small proportion of the total variability in the unweighted data. Using the weighted algorithm, at the interface between the two clusters “oxic” and “oxygen-limited,” mostly communities from endpoint sampling are found, regardless of the oxygen contents of the bottom water or added minerals (Figure 4A).

PCoAs were also performed on subsets of oxic (Figures 4C,D) and oxygen-limited (Figures 4E,F) treatments using both weighted and unweighted algorithms. Although each experimental setup of sediment core incubations had similar bacterial starter communities, community compositions changed over time in all treatments. However, these changes appeared either random, as in the oxic calcite and dunite treatments with no consistent separation across parallel incubations (Figures 4C,D) or, as in the oxygen-limited incubations, communities developed distinctly over time, but untreated experiments and mineral treatments clustered together (Figures 4E,F).

### 3.4 Bacterial community composition

**Figure 5:**
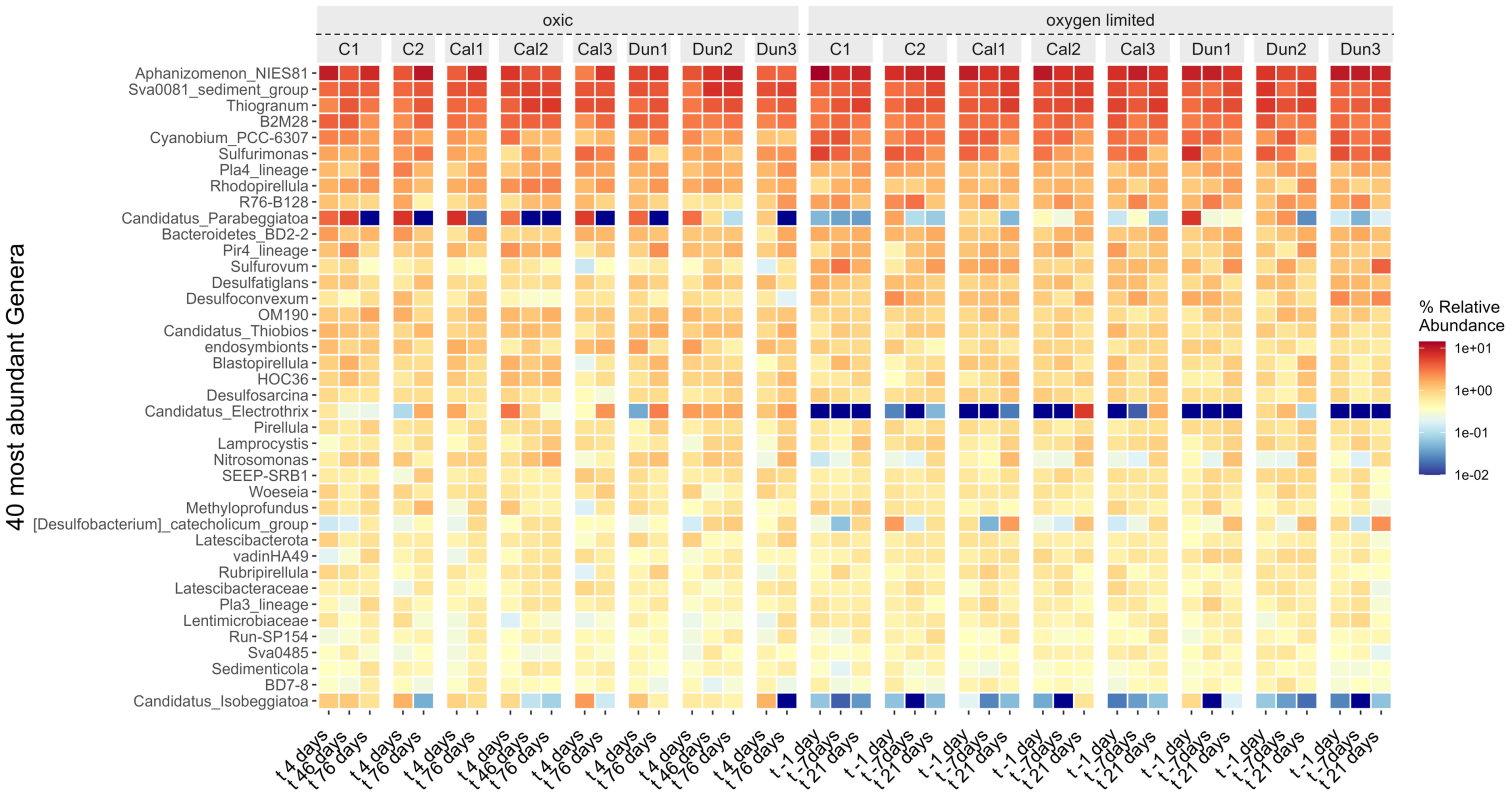
Heatmap showing the 40 most abundant genera in the 0–2 cm horizons of the oxic (left) and oxygen-limited (right) sediment core incubations. Samples were taken at three time points: for oxic incubations, t0 (−7 days before minerals were added), t2 (1 day before minerals were added) and t3 (end of experiments, 21 days after minerals were added); for oxygen-limited incubations, t0 (4 days after mineral addidion), t5 (46 days after mineral addition) and t6 (76 days after mineral addition). Controls (C1, C2) represent untreated incubations, Cal1-Cal3 are the calcite treatments, and Dun1-Dun3 are the dunite treatments. Samples were rarefied to the sample with the lowest read count (=31,500 reads per sample) prior to calculation of relative abundances. Colors in the heatmap indicate relative abundance (%) on a gradient from dark blue (low, 0.01) through yellow to dark red (high, 10).

All surface sediments were largely dominated by cyanobacterial *Aphanizomenon* NIES81 (*Nostocaceae*), potential sulfate-reducing members of the Sva0081 sediment group (*Desulfosarcinaceae)* and members of the potential sulfide-oxidizing *Thiogranum* (*Thiotrichaceae)* and B2M28 (*Gammaproteobacteria*). Also, various genera within the organotrophic *Pirellulaceae* were detected with key players including *Rhodopirellula, Blastopirellula*, *Pirellula*, and *Rubripirellula* which, together with an unspecified *Pirellulaceae* genus, make *Pirellulaceae* the most abundant family across all treatments (Figure 5, Supplementary Figures S4, S5). Around 25% of the bacterial community could not be assigned to any known phyla and were classified as unidentified taxa (Supplementary Figure S5). Their potential feedbacks to mineral addition remain enigmatic as they were excluded from statistical analyses.

**Figure 6:**
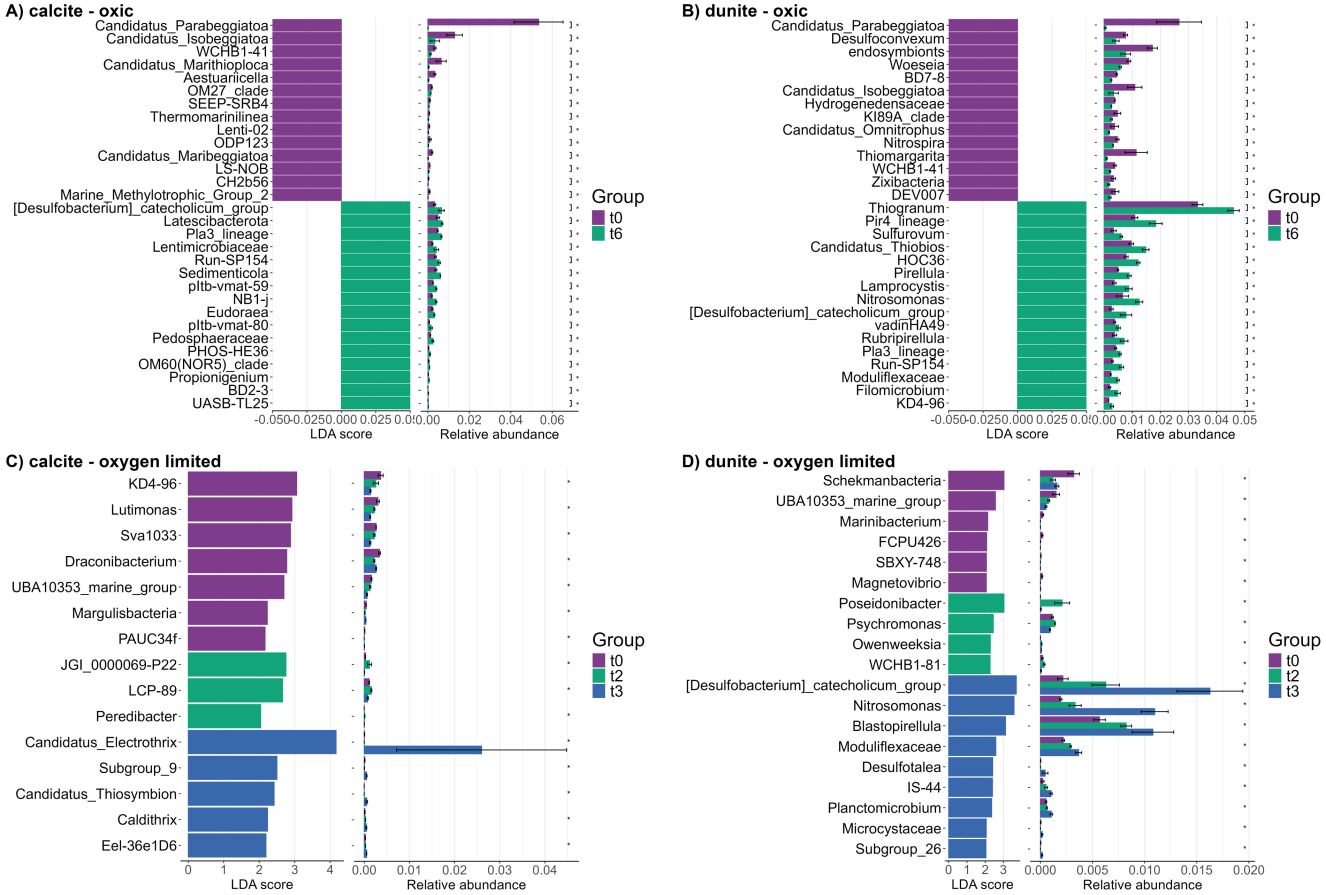
Differential abundance analyses (DAA) plots for subset data of calcite **(A,C)** and dunite **(B,D)** treated sediment cores incubated under oxic **(A,B)** versus oxygen-limited **(C,D)** bottom water conditions. *p*-values are indicated with asterisks, whereby the threshold of significance levels was set as * < 0.05, ** < 0.01 and *** < 0.001. Time points are color coded: t0 = 4 days (purple) and t6 = 76 days (green) for the oxic incubation; t0 = −7 days (purple), t2 = −1 day (green) and t3 = 21 (blue) for the oxygen-limited incubation. The analysis was performed using the linear discriminant analysis (LDA) effect size (LEfSe) method (Segata et al., 2011) in R with the microeco package (Liu et al., 2021) and sequence rarefaction of 31,500.

According to differential abundance analyses (DAA), unamended treatments under oxic or oxygen-limited water conditions did not give significant shifts of bacterial taxa during the experiments. In contrast, significant shifts in certain taxa were detected for mineral treatments kept under oxic or oxygen-limited bottom waters (Figures 6A–D). Under oxic incubation conditions relative abundance of members of the *Desulfobacterium catecholicum* group (*Desulfobacterota*), the Pla3 lineage (*Planctomycetota*) and the denitrifying genus Run-SP154 increased on a level of significance, independent of the type of mineral added, whereas *Candidatus* Parabeggiatoa and *Candidatus* Isobeggiatoa (both *Thiotrichaceae*) showed a marked decline (Figures 6A,B). After dunite addition with oxic overlying waters specific SOB became enriched including the genera *Thiogranum* (*Ectothiorhodoceae*), *Sulfurovum* (*Sulfurovaceae*) and *Candidatus* Thiobios (*Gammaproteobacteria*) (Figure 6B). Ammonia oxidizing bacteria (AOB) of the genus *Nitrosomonas* (*Nitrosomonadaceae*) were significantly enriched in dunite treatments, regardless of oxygen availability (Figures 6B,D).

Under oxygen-limited incubation conditions and amended calcite, the enrichment of *Candidatus* Electrothrix (cable bacteria performing long distance electron transport) after 21 days (t3) was most striking (Figure 6C). However, the associated standard deviation is high, reflecting natural variability among replicates, as cable bacteria enrichment was observed in only two out of the three parallel calcite-amended cores (0.02% in Cal1, 6.3% in Cal2 and 1.5% in Cal 3, Supplementary Figure S6). CARD-FISH analysis verified cable bacteria enrichment in the 0–2 cm sediment horizon of Cal2 under oxygen-limited bottom waters DSB706 signals were from cable bacteria-like filamentous cells, but also from non-cable bacteria rod-shaped single cells or cell aggregates (Supplementary Figures S7A–D). Cable bacteria-like filaments were thin, elongated cells (0.6 μm width and 5.0 μm length) and thick cells (1.9 μm wide and 3.5 μm long) indicating two cable bacteria morphotypes, positing at least one further cable bacterium type other than *Candidatus* Electrothrix in the sample. A possible unrecognized cable bacteria in Boknis Eck sediments has previously been suggested, since sediment pH profiles gave the typical cable bacteria pH excursions, but less than 1% of bacterial 16S rRNA genes could be assigned to *Candidatus* Electrothrix (Fuhr et al., 2023). The NON-EUB probe indicated no unspecific binding (Supplementary Figures S7E–H). Under dunite amendment and oxygen-limited waters representatives of the *Desulfobacterium catecholicum* group increased only in the dunite-amended core incubations but not when calcite was added, similar to the organotrophic *Blastopirellula* (*Pirellulacea*) (Figure 6D).

### 3.5 Correlation of community composition with sediment properties

**Figure 7:**
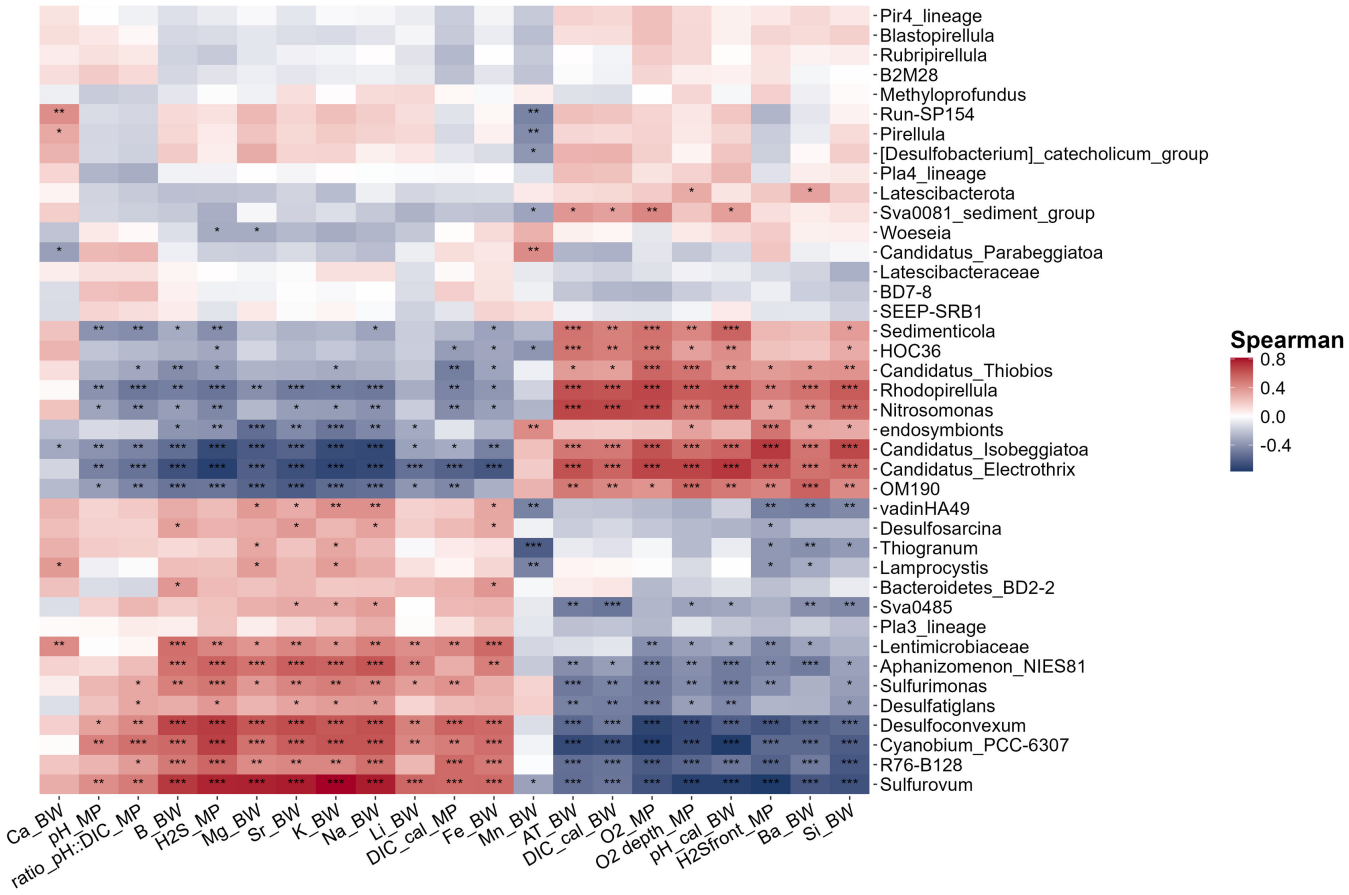
Spearman’s rank correlation coefficients between the relative abundances of the 40 most abundant bacterial genera (rarefied to 31,500 reads per sample) and measured environmental parameters across all samples. *p*-values were adjusted using the Benjamini-Hochberg false discovery rate (FDR). Significance levels are indicated by asterisks (*p*-value ≤ 0.05 = *, *p*-value ≤ 0.01 = **, *p*-value ≤ 0.001 = ***). Colors represent the strength and direction of the correlation (red = positive, blue = negative; range −1 to +1). Environmental parameters were measured or calculated (cal) for bottom water (BW) or derived from microprofile measurements (MP). Appreciations are as follows: AT_BW – total alkalinity in bottom water; DIC_cal_BW –dissolved inorganic carbon calculated for bottom water; pH_cal_BW – pH calculated for bottom water; DIC_cal_MP – dissolved inorganic carbon calculated from microprofile measurements of pH; H_2_S_MP – hydrogen sulfide based on microprofile measurments; O_2_ depth_MP – oxygen penetration depth based on microprofile measurments; H_2_Sfront_MP – depth of the hydrogen sulfide front based on microprofile measurments; ratio_pH: DIC_MP – ratio between pH and DIC.

**Figure 8:**
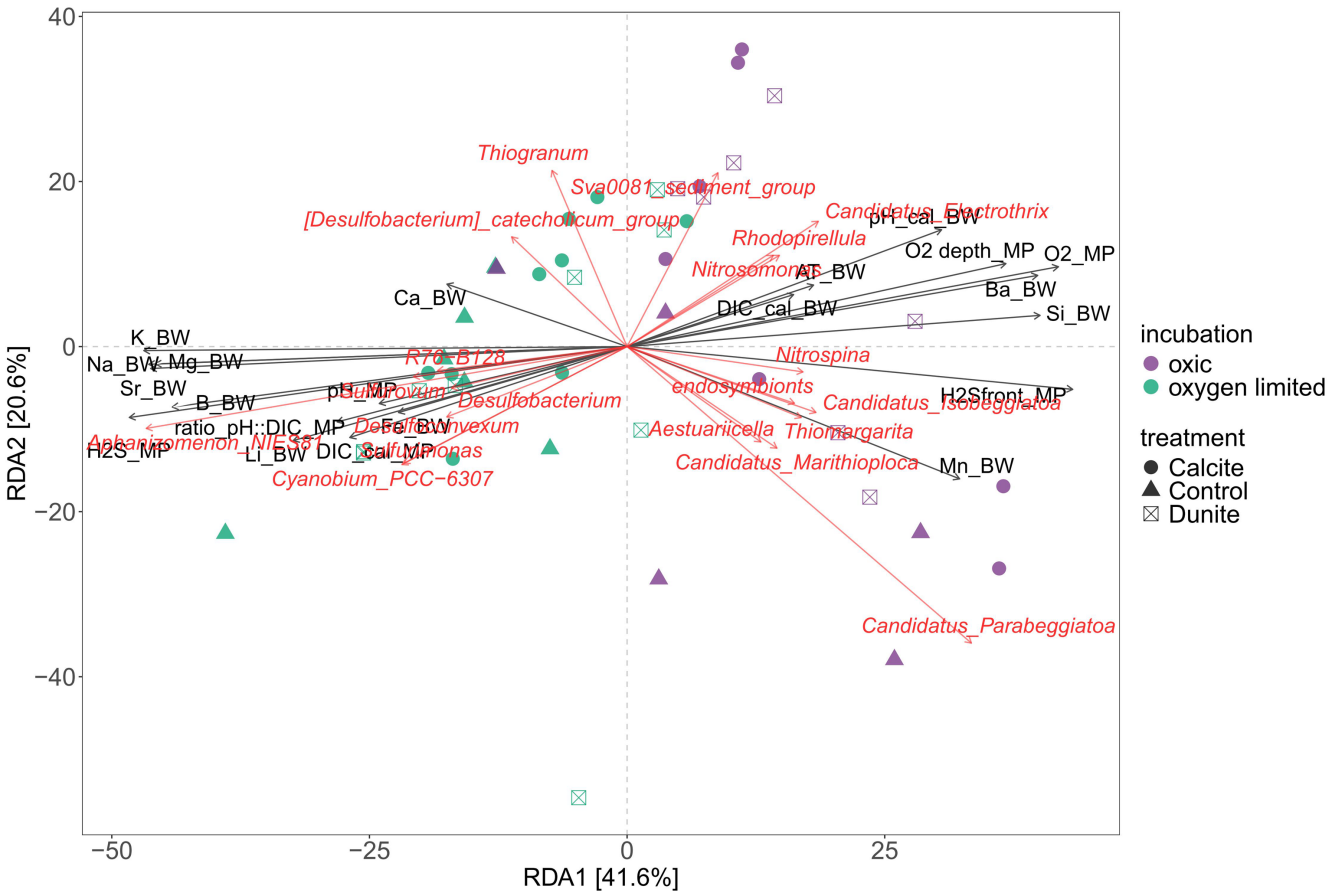
Redundancy analysis (RDA) plot representing the relationship between 16S rRNA amplicon abundances of the 20 most relevant bacterial taxa, bottom water (BW) chemistry and microprofile (MP) data of the top 2 cm sediment horizon. The parameters included for RDA explain 62% of taxon variability. Arrows pointing in the same direction indicate positive correlation, opposite directions indicate negative correlation. Arrow length reflects the strength of the variable’s association with sample difference. Abbreviations used are the same as those described in legend of Figure 7.

Overall, from five taxa that were shown to be dominant across all treatments, three exhibited no or comparatively few significant correlations with measured environmental parameters (Figure 6), suggesting a high degree of ecological robustness and limited sensitivity to short-term environmental variation. This includes members of the Sva0081 sediment group, *Thiogranum* and B2M28. The Sva0081 sediment group showed positive correlations on a level of significance with A_T_, O_2_, bottom water DIC and bottom water pH, and a negative correlation with Mn (Figures 7, 8). *Thiogranum* also displayed a significant negative correlation with dissolved Mn concentrations (*p* = 1.93 × 10^−05^), Si, Ba and the depth at which H_2_S was detectable with microelectrodes (H_2_S front), while showing a positive correlation with Mg and K. The B2M28 show no statistically significant relationship to any of the tested environmental parameters.

Several genera that exhibit significant correlations with nearly all tested environmental parameters (except Mn and Ca) were observed, suggesting that these taxa are highly responsive to the prevailing geochemical gradients. Based on the correlation patterns two groups can be distinguished (Figure 7). The first group shows positive correlations with A_T_ in bottom water (AT_BW), dissolved inorganic carbon (calculated for bottom water, DIC_cal_BW), oxygen (from microprofile measurements, O_2__MP), pH (calculated for bottom water, pH_cal_BW), the oxygen penetration depth (O_2_ depth_MP), silicate (Si_BW), the depth of the hydrogen sulfide front (H_2_S front_MP), and barium (Ba_BW), alongside negative correlations with hydrogen sulfide (from microprofile measurements, H_2_S_MP), boron (B_BW), magnesium (Mg_BW), potassium (K_BW), sodium (Na_BW), strontium (Sr_BW), the ratio between pH and DIC (based on microprofile measurements, ratio_pH: DIC_MP), pH (from microprofile measurements, pH_MP), lithium (Li_MP), dissolved inorganic carbone (calculated based on microprofile measurements, DIC_cal_MP), and total dissolved iron (Fe_BW). This group includes the genera OM190, *Candidatus* Electrothrix, *Rhodopirellula, Nitrosomonas* and *Candidatus* Thiobios. Most striking here, however, is *Candidatus* Electrothrix, exhibiting the strongest negative correlations with H_2_S, K, Sr., and Fe, and pronounced positive correlations with O_2_ and the onset of the anoxic zone (O_2_ depth_MP) (Figures 7, 8), underlining its role in oxygen-dependent sulfide and iron oxidation.

Compared to the first group, the second group shows an opposite correlation pattern. This group includes the genera *Sulfurimonas*, *Desulfatiglans*, *Aphanziomenon* NIES81, *Desulfoconvexum*, *Cyanobium* PCC-6307, R76-B128, and *Sulfurovum*. The latter stands out due to its strong positive correlations with H_2_S, Mg, K, Na, and Sr., as well as strong negative correlations with O_2_, the oxygen penetration depth (O_2_ depth_MP), and the position of the H_2_S front (Figures 7, 8), consistent with an adaptation to more reduced, sulfide-rich microenvironments.

## 4 Discussion

The concept of EBW relies on the idea that alkalizing minerals such as calcite or dunite are added to the seafloor, where they release alkalinity as they dissolve and thereby increase atmospheric CO_2_ uptake by the ocean. To evaluate the environmental implications of this approach, sediment core incubations with and without mineral addition were performed under contrasting oxygen conditions. The geochemical responses of these incubations have been comprehensively assessed in Fuhr et al. (2023, 2024), demonstrating that calcite amendments under oxygen-limited bottom waters produced the highest benthic A_T_ fluxes and thus the strongest apparent mCDR potential (Supplementary Figure S1). Building on this geochemical data assessment, the present study focusses on the microbial responses to EBW. Specifically, we have examined how mineral-driven A_T_ enhancement via dunite and calcite dissolution affected sediment microbial communities and how microbial taxa dynamics correlated with key geochemical parameters. 16S rRNA gene amplicons were generated from reverse-transcribed environmental RNA (cDNA) to specifically target the metabolically active bacterial community in the top 2 cm sediment horizons across all treatments. Overall, the bacterial community composition of the natural sediments closely resembled previous reports from Boknis Eck (Alampalli et al., under review; Perner et al., 2022), implying that the communities in the experiments are representative of this study site. Alpha diversity was evaluated to determine whether bacterial community diversity differed among incubation conditions and mineral treatments. The observed Shannon diversity was remarkably consistent across all samples, averaging 6.56 ± 0.2 (*n* = 43), and fell within the range previously reported for Boknis Eck sediments (Perner et al., 2022) and other organic-rich Baltic Sea sediments (Seidel et al., 2026). The absence of significant differences in Shannon diversity between incubation conditions and mineral treatments suggests that microbial richness and evenness remained largely stable throughout the experiment, despite the geochemical changes induced by mineral addition. Patterns of beta diversity were analyzed to resolve treatment- and incubation-specific differences in bacterial community structure. Principal coordinate analysis (PCoA) revealed a clear separation of samples primarily driven by oxygen availability in the overlying waters, indicating that redox regime and, potentially, seasonal differences at the time of sampling, represented the dominant structuring factors of the bacterial communities (Figures 4A,B). Mineral-specific effects were, by contrast, less pronounced, as evidenced by the lack of treatment specific clustering in the PCoA (Figures 4C–F). However, differential abundance analysis (DAA) identified specific taxa that contributed to community differentiation caused by alkaline mineral addition on a level of significance (Figure 6). Notably, no significant changes in taxa abundance were observed in the control incubations of oxic and oxygen-limited incubation over time, respectively. While this suggests limited temporal and redox-driven variability under the two experimental conditions, the results should be interpreted with caution given the limited replication in the control treatment (*n* = 2 due to technical constrains).

Mineral addition under oxic conditions was associated with a significant decline in relative abundances of members of *Beggiatoaceae* (sub-family within the *Thiotrichacea*), including *Candidatus* Parabeggiatoa and *Candidatus* Isobeggiatoa. In the calcite treatment, the relative abundance of *Candidatus* Parabeggiatoa decreased from 5.3% at t0 to 0.01% at t6 (99.8% decline), while *Candidatus* Isobeggiatoa decreased from 1.3% at t0 to 0.3% t6 (76.9% decline). Similar trends were observed in the dunite treatments, where relative abundances declined over the course of the experiment from 2.7 to 0.04% (98.5% decline of *Candidatus* Parabeggiatoa) and from 1.1 to 0.3% (72.7% decline *Candidatus* Isobeggiatoa), respectively. *Beggiatoaceae* are chemolithotrophic bacteria that oxidize sulfide to elemental sulfur using oxygen or nitrate (NO_3_^−^) as a terminal electron acceptor (Teske and Salman, 2013), making them characteristic inhabitants of sulfidic sediments such as those at Boknis Eck (Perner et al., 2022; Dale et al., 2011). Here, they are of particular ecological relevance as they contribute to the regulation of sulfide (H_2_S) concentrations (Preisler et al., 2007). By oxidizing H_2_S, they prevent release of H_2_S from the sediment into the overlying water column, thereby reducing the toxic effects of sulfide for higher organisms (Teske and Salman, 2013; Schulz and Jørgensen, 2001). Although less pronounced and not statistically significant compared to the calcite treatments, downward trends in the relative abundance of *Candidatus* Parabeggiatoa and *Candidatus* Isobeggiatoa were also observed in the control and dunite treatments (Figure 5), suggesting that the experimental conditions independent of mineral addition, may have imposed a general growth disadvantage for *Beggiatoaceae* spp. While *Candidatus* Parabeggiatoa showed a significant correlation with Mn and Ca concentrations in the overlying water (Spearman’s rank correlation, r_s_ = 0.42, *p* = 5.18 · 10^−3^ and r_s_ = −0.35, *p* = 0.021, respectively), *Candidatus* Isobeggiatoa significantly correlated with almost all tested environmental parameters (*p* < 0.05), with the exception of Mn (r_s_ = 0.23, *p* = 0.14) (Figure 7). This indicates that *Candidatus* Isobeggiatoa is more sensitive to environmental changes induced by redox regime and mineral addition compared to *Candidatus* Parabeggiatoa. The similar orientation of both genera along the same RDA gradient defined by the H_2_S front and bottom water Mn (Figure 8), however, likely reflects their response to a shared redox-related environmental gradient as a main driver of their abundance. The decrease of *Beggiatoaceae* may have opened an ecological niche for other SOB. Consistent with this interpretation, various SOB taxa increased in relative abundance, including *Thiogranum* (*Ectothiorhodoceae*), *Sulfurovum* (*Sulfurovaceae*), *Candidatus* Thiobios (*Gammaproteobacteria*) and HOC36 (LEfSe; *n* = 3, all *p* < 0.05) (Figure 6B). This pattern indicates a potential restructuring within the sulfide-oxidizing microbial community, in which different SOB taxa may partially replace the functional role previously dominated by *Beggiatoaceae*. This is further supported by stable H_2_S concentrations in the top 2 cm horizon (Supplementary Figure S7 in Supplementary material to Fuhr et al. (2023). However, when calcite was added under oxic bottom water conditions, the decline of *Beggiatoacaea* was not compensated by an increase in other SOB taxa. Instead, an enrichment of organotrophic taxa was observed including *Latescibacterota*, Pla3_lineage and *Lentimicrobiaceae* (Figure 6A). Calcite addition under oxic bottom waters may thus directly or indirectly limit the growth of other SOB taxa while providing a relative advantage to organotrophic microorganisms. However, the decline in *Beggiatoaceae* spp. and the lack of a compensatory increase in other SOB taxa are not consistently reflected in H_2_S profiles, as only slight increases in H_2_S concentrations in the top 2 cm horizon were observed in Cal1 and Cal2, whereas H_2_S concentrations in Cal3 decreased (Supplementary Figure S7 in Supplementary material to Fuhr et al. (2023).

*Candidatus* Electrothrix (*Desulfobulbaceae*) appears particularly responsive to calcite amendments under oxygen-limited bottom water conditions (LEfSe; *n* = 3, *p* = 0.03, LDA score = 4.2) (Figure 6C, Supplementary Figure S6). Members of the genus *Candidatus* Electrothrix are widespread in the Baltic Sea and can greatly modify porewater chemistry, even at low abundances (Marzocchi et al., 2020; Hermans et al., 2019). These filamentous multicellular bacteria belong to the so-called group of cable bacteria and as such they allow for vertical long-distance electron transport over centimeter distance, coupling sulfide oxidation in the anoxic zone with oxygen reduction at the sediment surface (Marzocchi et al., 2014; Nielsen et al., 2010; Pfeffer et al., 2012). Subsurface sulfide oxidation by anodic cable bacteria cells releases 10 moles of protons per mole of sulfide (H_2_S) oxidized (Burdorf et al., 2024). This porewater acidification leads to dissolution of iron sulfides (FeS) and Ca/Mn carbonates, mobilizing Fe, Ca and Mn, which diffuse upwards, where higher pH and oxygen availability facilitate Fe/Mn-oxide and Mg-calcite precipitation (Geerlings et al., 2019 and references therein). However, porewater acidification induced by cable bacteria may be counteracted by the level of the sediment pH buffering capacity, i.e., primarily carbonate dissolution (Rao et al., 2016; Burdorf et al., 2024). This may affect the availability of ferrous Fe (Fe^2+^) and thus Fe-oxide formation. The extent of Fe-oxide precipitation in low-oxygen systems is an important consideration because Fe-oxides efficiently bind free sulfide and thus act as a barrier to sulfide escaping from sediments during seasonal anoxia (Seitaj et al., 2015). By contrast, calcite addition to carbonate-rich sediments would increase sediment buffer capacity and thus counteract cable bacteria acidification to a certain degree, thus essentially making less iron available for near surface Fe-oxide precipitation. This could weaken the potential sedimentary barrier for sulfide effluxes during seasonal anoxia (Seitaj et al., 2015; Burdorf et al., 2024). The response of *Candidatus* Electrothrix was inconsistent across the three parallel calcite treated cores (0.02% in Cal1, 6.3% in Cal2 and 1.5% in Cal 3, Supplementary Figure S6) incubated under oxygen-limited water conditions (Supplementary Figure S6), which likely reflects temporary oxygen intrusion into Cal2 and Cal3, but not in Cal1 (Figure 2, right panel). Since no cable bacteria growth was observed in the control (C2) and the dunite treatments (Dun1 and Dun2) despite temporary exposure to oxygen during incubation, it can be concluded that the addition of calcite stimulates the growth of cable bacteria under periodically low-oxic water conditions. Cable bacteria are capable of autotrophic carbon fixation via the Wood-Ljungdale pathway and encode periplasmatic carbonic anhydrases (CAs) on their genomes (Teske, 2019; Kjeldsen et al., 2019). CAs (EC 4.2.1.1)) catalyze the reversible interconversion between HCO_3_^−^ and CO_2_ and can enhance CO_2_ fixation in autotrophic microbes (Kupriyanova et al., 2017). In the context of EBW, where mineral alkalinity is added to the seafloor to convert CO_2_ to HCO_3_^−^ (Dale et al., 2024), stimulated microbial CA activity might affect the effectiveness of OAE. Under the given experimental conditions, the genus *Candidatus* Electrothrix was highly responsive to chemical dynamics, showing significant correlations with all measured environmental parameters except Mn and Ca (Figure 7). The strongest positive correlation was observed with pH (Spearman’s rank correlation, r_s_ = 0.71, *p* = 9.83 · 10^−8^), followed by O_2_ (r_s_ = 0.67, *p* = 1.06 · 10^−6^), O_2_ penetration depth (r_s_ = 0.67, *p* = 9.13 · 10^−7^), A_T_ (r_s_ = 0.62, *p* = 7.23 · 10^−6^), H_2_S front (r_s_ = 0.60, *p* = 1.80 · 10^−5^) and DIC (r_s_ = 0.56, *p* = 9.28 · 10^−5^). In the RDA plot, *Candidatus* Electrothrix showed an orientation similar to *Nitrosomonas* and *Rhodopirellula* (Figure 8), reflecting comparable responses to geochemical gradients linked to alkalizing mineral addition, but also revealed links between microbial dynamics and geochemical changes in the incubation.

Members of the genus *Nitrosomonas* (family *Nitrosomonadacaea*) were enriched in both dunite treatments, exhibiting a 1.9-fold increase in relative abundance under oxic conditions (0.7 to 1.3%) and a 5.5-fold increase under oxygen-limited conditions (0.2 to 1.1%) (Figures 6B,D). At the species level, this included unassigned *Nitrosomonas* species as well as relatives of the species *Nitrosomonas cyrotolerance*. Although *Nitrosomonas* accounted for less than 2% of the total microbial community in the present sediment core incubations, previous studies have shown that even low-abundance *Nitrosomonas* populations can exhibit high transcriptional and metabolic activity (Cholet et al., 2024). Cultivated representatives of *Nitrosomonas* are obligate chemolithoautotrophs that oxidize ammonia (NH_3_) to nitrite (NO_2_^−^), which can be further oxidized to nitrate (NO_3_^−^) by nitrite oxidizing bacteria, completing the nitrification (Prosser et al., 2014). Enhanced nitrogen availability in coastal environments commonly promotes eutrophication, which can ultimately result in oxygen depletion and the development of water column hypoxia or anoxia (Gruber and Galloway, 2008). Nitrogen removal processes such as microbial denitrification and anerobic ammonium oxidation (anammox) counteract this accumulation by converting reactive nitrogen species (NO_2_^−^; NO_3_^−^) into dinitrogen gas (N_2_) (Damashek et al., 2014). In this context, nitrifying organisms including *Nitrosomonas*, play a major role in the nitrogen cycle as ammonia oxidation regulates the availability of both NO_2_^−^ and NO_3_^−^ that serve as substrates for downstream nitrogen loss pathways (Seitzinger et al., 2006; Cholet et al., 2024; Wang et al., 2025). The observed increase of *Nitrosomonas* following dunite addition could therefore represent a beneficial side effect, contributing to mitigate eutrophication of the Baltic Sea (Kuss et al., 2026; Murray et al., 2019). Previous studies have shown that ammonia-oxidizing microbes can adapt to acidification by increasing their substrate affinity as a compensatory response (Tong et al., 2026). However, in the sediment cores collected in October 2022 for the oxygen-limited incubation experiment, *Nitrosomonas* abundances tended to increase not only in the dunite-treatment, but to some extent across all sediment cores (Figure 5). Thus, conditions in the oxygen-limited incubation appear to promote the growth of *Nitrosomonas*, while the dunite addition seems to provide an additional growth boost. *Nitrosomonas* spp. are classically considered aerobic chemolithoautotrophic ammonia oxidizers, although several species have been shown to perform nitrifier denitrification under reduced oxygen concentrations, using NO_2_^−^ as terminal electron acceptor to form gaseous products like dinitrogen (N^2^), nitrous oxide (N_2_O) and nitric oxide (NO), which are ultimately released from the ecosystem (Koops et al., 1991; Herbert, 1999; Schmidt et al., 2004). This metabolic strategy likely explains their persistence in the present sediment cores incubated under oxygen-limited bottom water conditions. Across the measured environmental gradients, *Nitrosomonas* abundance correlated with almost all tested parameters, with the strongest correlation observed for A_T_ (Spearman’s rank correlation, r_s_ = 0.67, *p* = 1.10 · 10^−6^), dissolved inorganic carbon (r_s_ = 0.66, *p* = 1.57 · 10^−6^), and bottom-water oxygen (r_s_ = 0.65, *p* = 2.57 · 10^−6^). The positive correlation with oxygen is consistent with a possible change in the metabolism from ammonia oxidation to nitrifier denitrification due to oxygen availability, while the correlations with a A_T_ and DIC may reflect the altered carbonate chemistry induced by mineral weathering and the autotrophic lifestyle of *Nitrosomonas*, which relies on the fixation of inorganic carbon.

## 5 Conclusion

To our knowledge, this study provides the first assessment of the effects of enhanced benthic weathering (EBW) as a marine carbon dioxide removal (mCDR) strategy on benthic microbial communities. Laboratory-based sediment core incubations demonstrated that the addition of calcite and dunite induced significant shifts in the relative abundances of specific bacterial taxa, while the overall community structure and diversity remained largely stable. These findings suggest that microbial responses to EBW are primarily expressed through changes in selected community members rather than through a large-scale restructuring of the bacterial community.

The ecological and biogeochemical consequences of these taxonomic shifts, however, remain to be resolved. The hypothesized feedbacks derived from the chemistry of overlying bottom waters and sediment porewaters should therefore be validated through complementary analyses, including transcriptomics and microbial metabolic rate measurements, including, e.g., sulfate reduction, oxygen consumption, and carbon assimilation.

While sediment core incubations provide a controlled framework for assessing microbial responses to mineral addition, they cannot fully capture the spatial heterogeneity, biological interaction, and seasonal variability of natural coastal sediments. Consequently, the long-term ecosystem effects of EBW remain uncertain. Nevertheless, since EBW has proven to be an effective and cost-efficient strategy for mCDR (Fuhr et al., 2024; Dale et al., 2024), our results provide an important basis for future field-scale studies aimed at evaluating the ecological feasibility and environmental consequences of alkaline mineral deployment on the seafloor.

## Data Availability

The 16S rRNA gene sequences are available in the NCBI GenBank under accession number PRJNA1192588. The geochemical data (Fuhr et al., 2023, 2024) are deposited in PANGEA under the following DOIs: 10.1594/PANGAEA.974076, 10.1594/PANGAEA.974077, 10.1594/PANGAEA.974078, 10.1594/PANGAEA.974079, 10.1594/PANGAEA.977247, 10.1594/PANGAEA.977249, and 10.1594/PANGAEA.977250.

## Footnotes

1
https://www.R-project.org/
