# Supplementary material for: Microbial responses to ocean alkalinity enhancement in seasonally hypoxic coastal sediments
Source: Front Microbiol. 2026 Jun 23;17:1854407. doi: 10.3389/fmicb.2026.1854407 (PMC13337938; doi:10.3389/fmicb.2026.1854407)
Supplement: Supplementary file 1 [file Data_Sheet_1.pdf]

Supplementary Information to

Microbial Responses to Ocean Alkalinity Enhancement in Seasonally Hypoxic Coastal Sediments

Stefanie Böhnke-Brandt[1*], Rebecca Bährle-Wunsch[1], Michael Fuhr[2], Andrew W. Dale[2], Janina Fuß[3], Sonja Geilert[4] and Mirjam Perner[1*]

[1]Geomicrobiology, GEOMAR Helmholtz Centre for Ocean Research Kiel, Kiel, Germany

[2]Benthic Biogeochemistry, GEOMAR Helmholtz Centre for Ocean Research Kiel, Kiel, Germany

[3]Competence Centre for Genomic Analysis (CCGA), Institute of Clinical Molecular Biology (IKMB), Kiel University, Kiel, Germany

[4] Department of Earth Sciences, Utrecht University, Utrecht, The Netherlands

*** Correspondence:**
Mailing Address: GEOMAR Helmholtz Centre for Ocean Research Kiel, Geomicrobiology, Wischhofstraße 1-3, 24148 Kiel, Germany. E-mail: sboehnke-brandt@geomar.de and mperner@geomar.de

Supplementary Figures

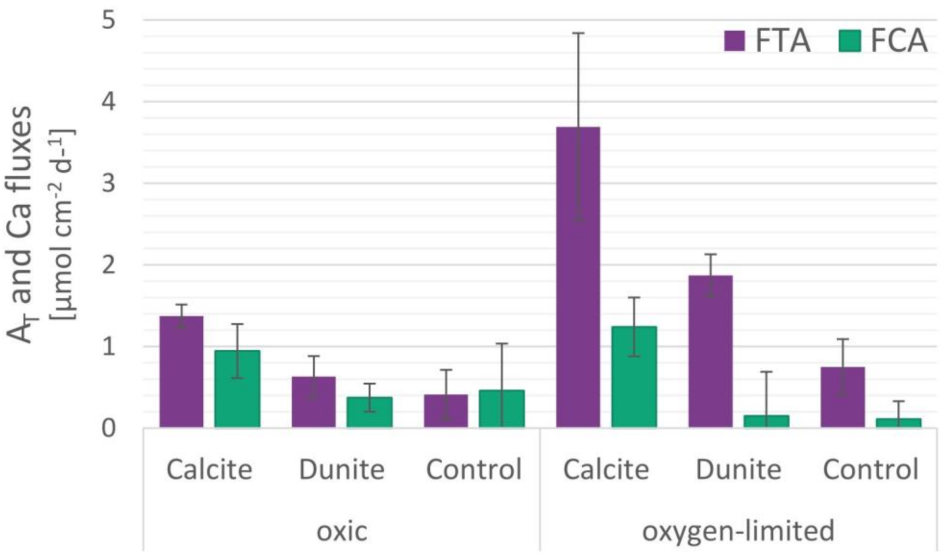

Supplementary Figure S1: Average A_T (total alkalinity) and Ca (calcium) fluxes from oxic and oxygen-limited incubations across different treatments, calculated over the last five days of the experiments.

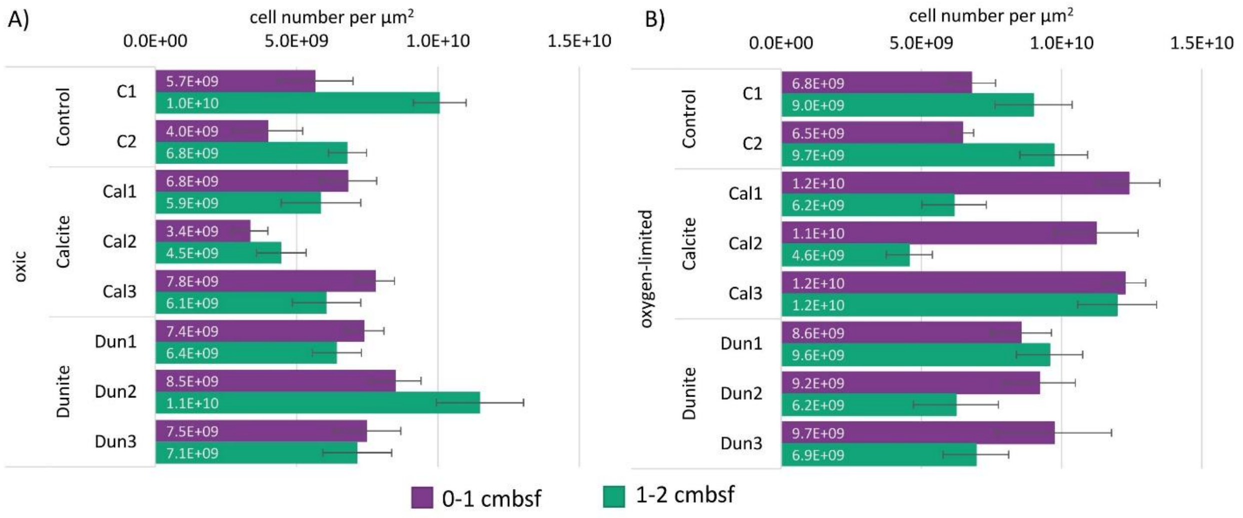

Supplementary Figure S2: Total cell numbers determined via Dapi. Cell numbers for sediment depth layers 0-1cmbsf (purple) and 1-2 cmbsf (green) are shown for individual replicates within a treatment of (A) oxic and (B) oxygen-limited incubations. Error bars indicate technical standard deviations based on cell distribution across the filter. Total cell counts represent the sum of cells recovered from pore water and cells detached from sediment particles via ultrasonication.

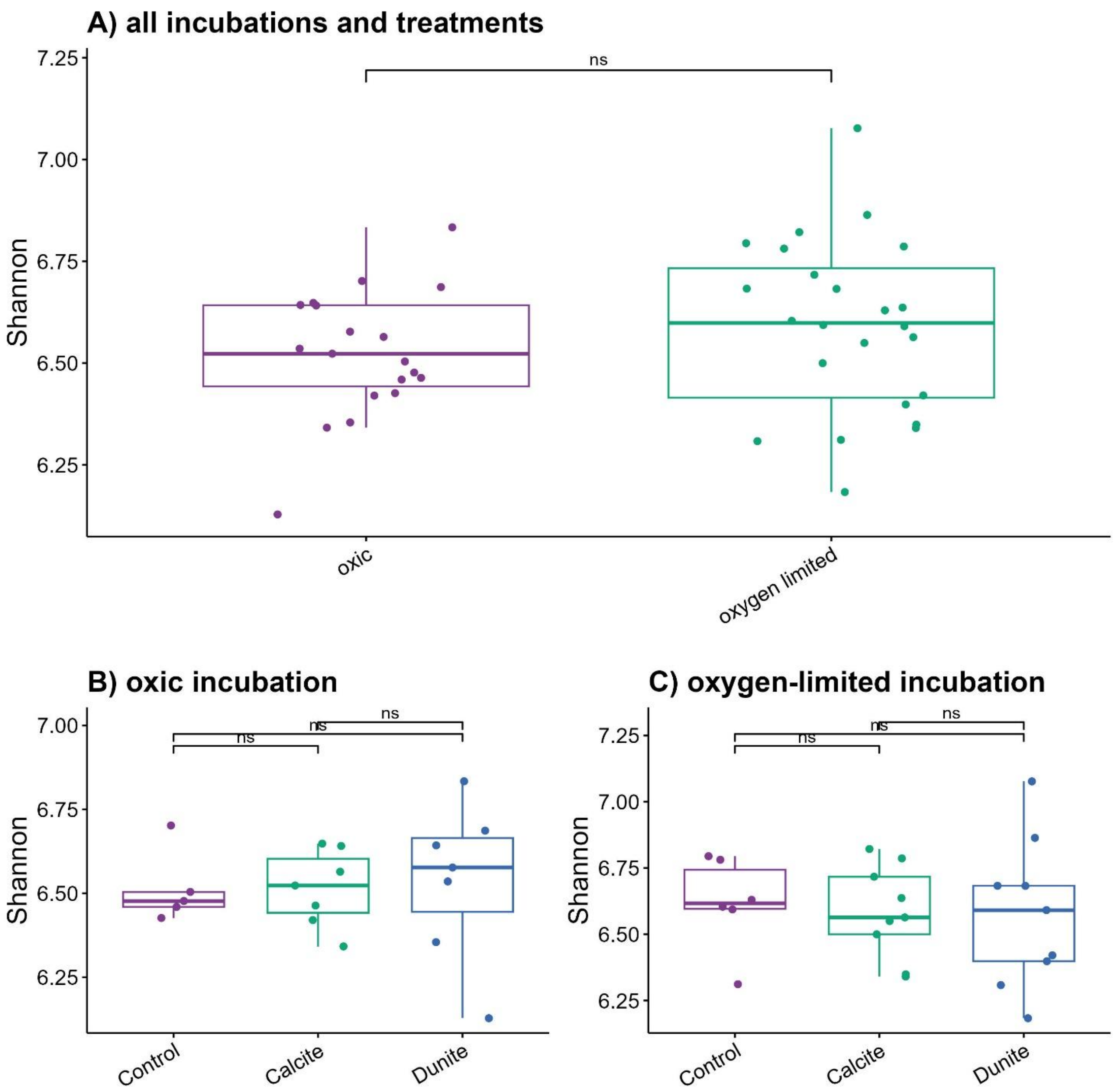

Supplementary Figure S3: Shannon diversity of bacterial communities is shown A) across all incubation conditions and treatments as well as for subsets of B) oxic and C) oxygen-limited incubations. Individual data points represent biological replicates. Wilcoxon rank-sum tests ($p < 0.05$) was used to assess significant differences between groups; ns indicates not significant.

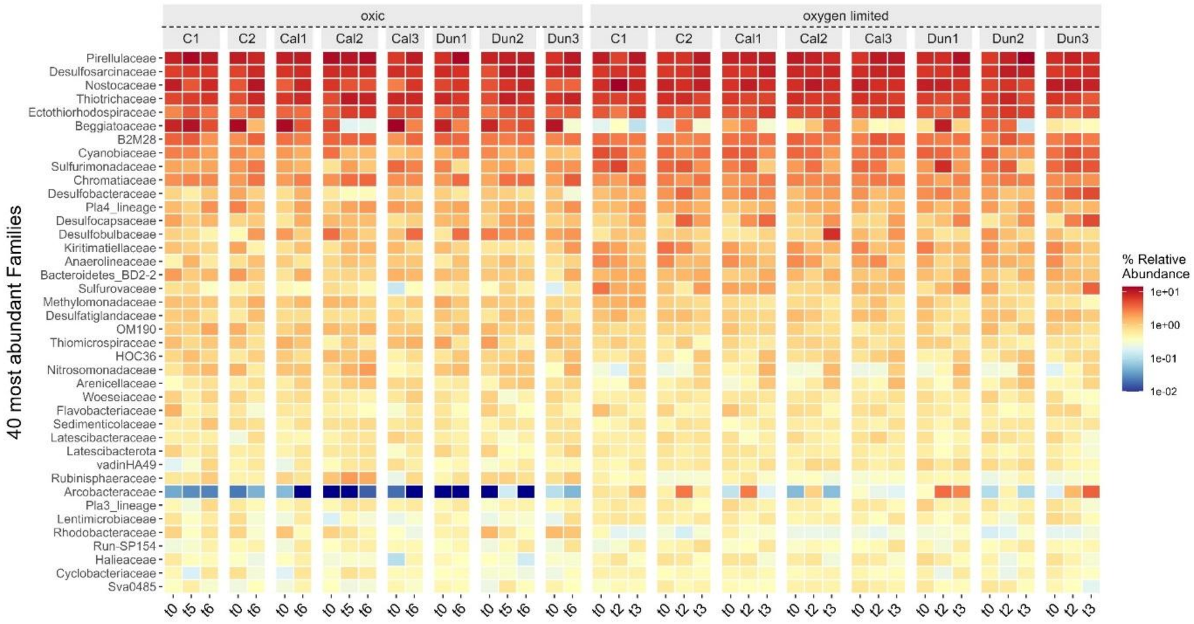

Supplementary Figure S4: Heatmap depicting the 40 most abundant families based on bacterial 16S rRNA gene amplicons. Relative abundances for the 0-2 cm horizons of the oxic (left) and oxygen-limited (right) sediment core incubations are shown. Samples were taken at three time points: for oxic incubations, t0 (7 days before minerals were added), t2 (1 day before minerals were added) and t3 (end of experiments, 21 days after minerals were added); for oxygen-limited incubations, t0 (4 days after mineral addidion), t5 (46 days after mineral addition) and t6 (76 days after mineral addition). Controls (C1, C2) represent untreated incubations, Cal1-Cal3 are the calcite treatments, and Dun1-Dun3 are the dunite treatments. Samples were rarefied to the sample with the lowest read count (=31500 reads per sample) prior to calculation of relative abundances. Colors in the heatmap indicate relative abundance (%) on a gradient from dark blue (low, $1e^{-02}$) through yellow to dark red (high, $1e^{01}$).

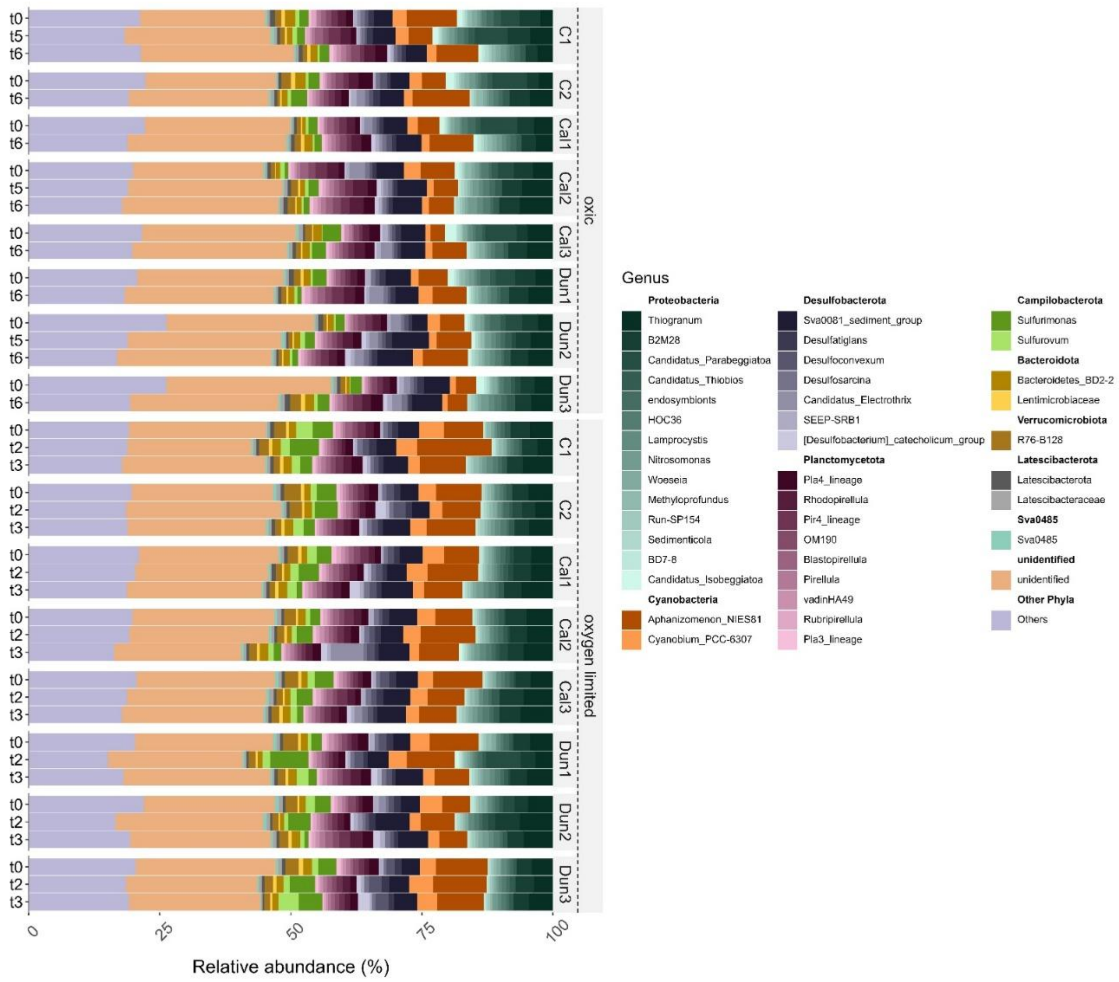

Supplementary Figure S5: Taxonomic composition of bacterial communities based on 16S rRNA amplicon sequence data. The relative abundance of bacterial taxa at the genus level in the 0-2cm horizon of the oxic (left) and oxygen-limited (right) sediment core incubations is shown for controls (C1, C2), representing untreated incubations, calcite amendments (Cal1, Cal2, and Cal3) and dunite amendments (Dun1, Dun2, and Dun3). The 40 most abundant genera are show. All other bacterial genera are grouped under "Others" (lilac). The left x-axis gives the sampling time points: For oxic incubations, t0 (-7 days before minerals were added), t2 (1 day before minerals were added) and t3 (end of experiments, 21 days after minerals were added); and for oxygen-limited incubations, t0 (4 days after mineral addidion), t5 (46 days after mineral addition) and t6 (76 days after mineral addition).

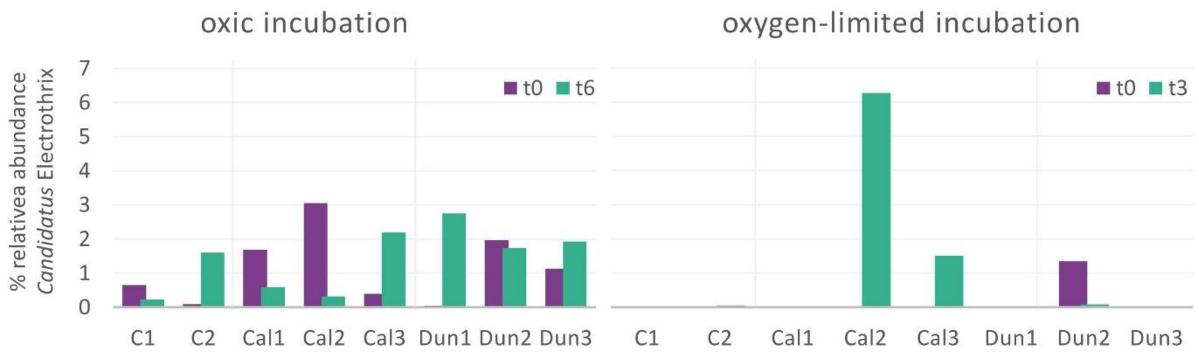

Supplementary Figure S6: Relative proportion of 16S rRNA gene amplicons related to *Candidatus* Electrothrix. Data is shown from the 0-2 cm horizons of the sediment core incubations for the oxic (left) and the oxygen-limited (right) incubation. Timepoints are defined as follows: for oxic incubations, t0 = 7 days before mineral addition and t3 = end of the experiments, 21 days after minerals were added; for oxygen-limited incubations, t0 = 4 days after mineral addidion and and t6 = 76 days after mineral addition. Controls (C1, C2) are the untreated incubations, Cal1, Cal2 and Cal3 are the calcite treatments and Dun1, Dun2, and Dun3 are the dunite treatments.

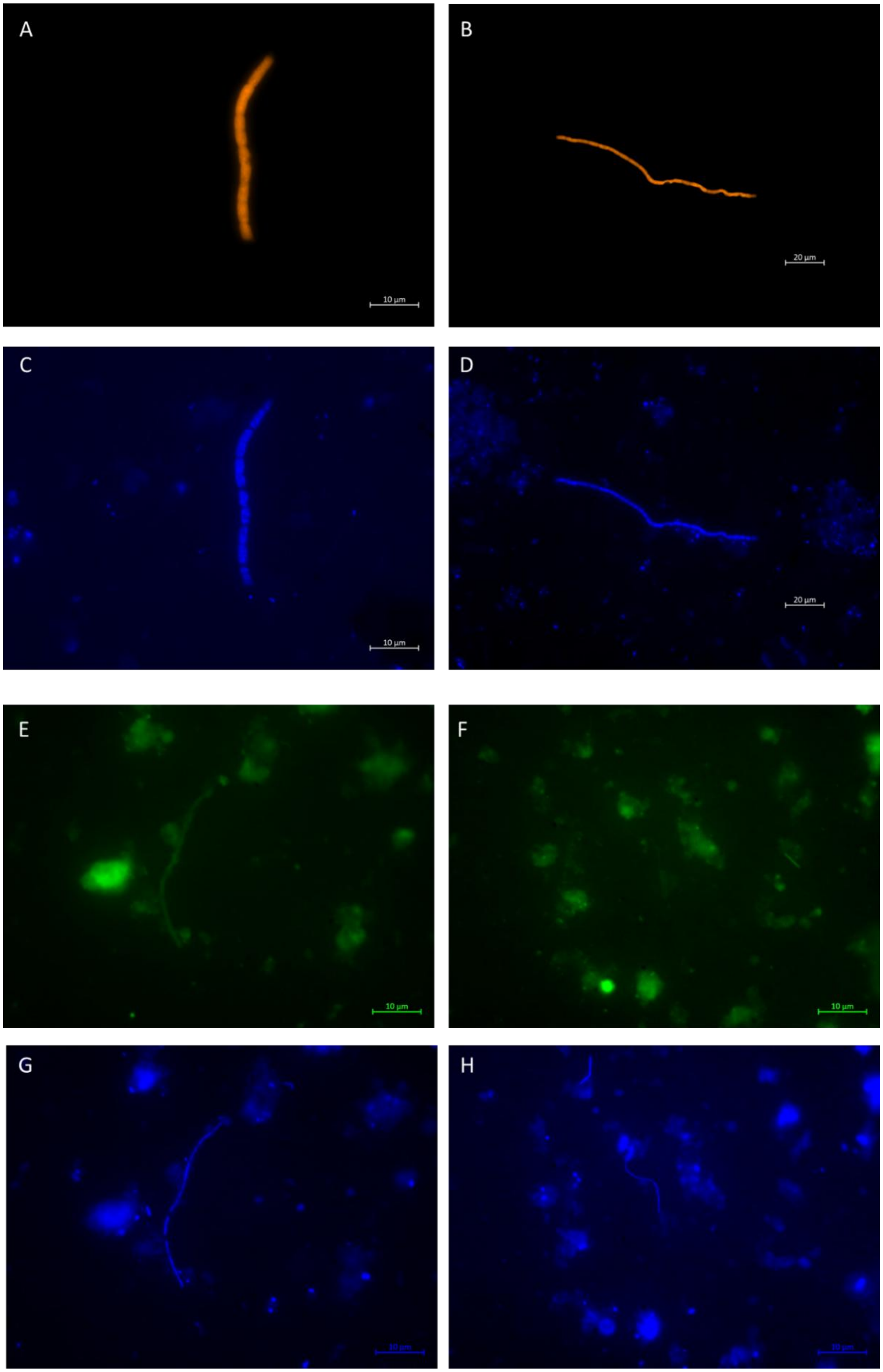

Supplementary Figure S7: (A, B) DSB706 and (E, F) NON-EUB probe signals and (C, D, G, H) respective DAPI stained cells for the Ca2 treatment at the end of the oxygen-limited incubation experiment.
